# Common barriers and enablers to the use of non-drug interventions for managing common chronic conditions in primary care: an overview of reviews

**DOI:** 10.1186/s12875-024-02321-8

**Published:** 2024-04-06

**Authors:** Hannah Greenwood, Alexandra R. Davidson, Rae Thomas, Loai Albarqouni

**Affiliations:** 1 Institute for Evidence-Based Healthcare, Faculty of Health Sciences & Medicine, Bond University, Gold Coast, Australia; 2 Faculty of Health Sciences & Medicine, Bond University, Gold Coast, Australia; 3 Tropical Australian Academic Health Centre, Townsville, Australia

**Keywords:** Overview of reviews, Primary health care, CFIR, TDF, Chronic disease, Implementation science, Non-pharmacological interventions, Nutrition therapy, Physical therapy modalities, Psychotherapy

## Abstract

**Background:**

Non-drug interventions are recommended for chronic condition prevention and management yet are underused in clinical practice. Understanding barriers and enablers to using non-drug interventions may help implement non-drug interventions in primary care. We aimed to conduct an overview of reviews to identify and summarise common barriers and enablers for using non-drug interventions for common chronic conditions in primary care.

**Methods:**

We included qualitative and quantitative reviews that used systematic process or methods to examine barriers and enablers to using non-drug interventions for chronic condition prevention and management in primary care settings. We searched 5 electronic databases (PubMed, Cochrane Database of Systematic Reviews, EMBASE, PsycInfo and CINAHL) from inception to September 2022. Two authors independently screened reviews. One author extracted and deductively coded data to Consolidated Framework of Implementation Research (CFIR) (and where relevant, Theoretical Domains Framework [TDF]). A second author validated 10% of extracted data and coding. Data was synthesised thematically using CFIR and TDF. One author assessed the methodological quality of included reviews using a modified AMSTAR 2 tool, with 10% validated by a second author. We assessed overlap between primary studies in included reviews.

**Results:**

From 5324 records, we included 25 reviews, with data predominately from patients. Overall, 130 subthemes (71 barrier and 59 enabler) were identified across 4 CFIR domains (Innovation, Outer Setting, Inner Setting, and Individuals), and all TDF domains. Common barrier and enabler subthemes were identified for CFIR constructs of Innovation Adaptability, Innovation Cost, Innovation Relative Advantage, Local Attitudes, External Pressure, Local Conditions, Relational Connections, Available Resources, and Access to Knowledge and Information. For TDF domains, important barrier and enabler subthemes were identified for Knowledge, Skills, Environmental Context and Resources, Beliefs about Consequences, Reinforcement, and Emotion.

**Conclusions:**

We synthesised reviews to provide new insight into common barriers and enablers for using non-drug interventions to prevent and manage chronic conditions in primary care. The factors identified can inform the development of generalisable implementation interventions to enhance uptake of multiple non-drug interventions simultaneously.

**Trial Registration:**

This study was registered in PROSPERO (CRD42022357583).

**Supplementary Information:**

The online version contains supplementary material available at 10.1186/s12875-024-02321-8.

## Background

Chronic health conditions are a major health burden, attributed to nearly three-quarters of all deaths annually [1, 2], and rates are rising [3]. Primary care services play a key role in chronic condition management, particularly through treatment and risk factor prevention and modification [4, 5]. Non-drug interventions (NDIs; also called lifestyle, or non-pharmacological interventions), such as dietary strategies, exercise, physical therapies, and psychological therapies, are frequently recommended in chronic condition prevention guidelines [6], and condition-specific management guidelines [7–10]. For example, international clinical practice guidelines for the management of osteoarthritis routinely recommend lifestyle and non-drug management options as first-line treatment [7–9, 11].

Despite this, observational evidence from the United States and United Kingdom suggests that patients with chronic conditions do not always receive lifestyle advice, when appropriate [12–15]. Analysis of the National Health and Nutrition Examination Survey (NHANES) from 2011 to 2016 show the proportion of patients in the United Kingdom with chronic conditions who receive advice varies, with patients with type 2 diabetes most likely to receive advice (56.5%), while patients with hypertension or high cholesterol received advice less often (31.4 to 27% respectively) [13]. Interestingly, lifestyle advice was rarely provided to adults without chronic conditions in the normal weight range (1–9%), despite being a known preventative strategy to reduce incidence of chronic conditions [13]. Subsequent analysis of NHANES data from 2015 to 2018 shows that receiving lifestyle advice is associated with higher likelihood of weight loss, increased physical activity, and reduced dietary sodium and fat intake, suggesting advice provision is associated with a reduction in risk factors [15].

The underuse of effective NDIs [16, 17] suggest factors inhibit their use in practice. Existing reviews of barriers and enablers often focus on specific stakeholders, interventions, or conditions. For example, a 2021 systematic review of primary care clinicians’ perceived barriers and enablers to dietary management of people with type 2 diabetes reported barriers including limited time for staff training, limited dietary knowledge, and lack of confidence in discussing dietary advice [18, 19]. While it is useful to understand condition or context-specific factors to implementing dietary management interventions, they may not generalise to different health conditions or interventions (i.e., we do not know whether clinician’s lack of confidence to discuss NDIs is unique to dietary advice interventions, or applies to other NDIs, such as physical activity advice). To address underuse of effective NDIs in primary care, understanding common reasons why clinicians and patients do or do not use effective NDIs more routinely is required.

To enable broader NDI implementation or to target multiple NDIs simultaneously, identifying and summarising the common factors for using or not using NDIs for chronic conditions is needed. Therefore, we aimed to conduct an overview of reviews to identify and summarise common barriers and enablers for using non-drug interventions for common chronic conditions in primary care. To encapsulate different domains for implementation and to ground our analysis in theory, we examined the results using the Consolidated Framework of Implementation Research (CFIR) and the Theoretical Domains Framework (TDF) [20].

## Methods

We prospectively developed a protocol for this overview and registered it in PROSPERO (CRD42022357583) and published it on Open Science Framework [21]. The Cochrane Handbook on Systematic Reviews of Interventions guided the study methods (“Overview of Reviews” chapter V) [22], and we reported results according to Preferred Reporting Items for Overview of Reviews (PRIOR) guidelines (see Additional File 1) [23]. Systematic review automation tools were used to facilitate deduplication, screening, and dispute resolution [24, 25].

### Eligibility criteria

We included full-text articles fulfilling Sample, Phenomenon of Interest, Design, Evaluation, Research type (SPIDER) criteria (Table 1) [26].

**Table 1:** SPIDER inclusion and exclusion criteria

SPIDER criteria	Inclusion	Exclusion
Sample	Primary care clinician^a^ (including primary allied health) referred the intervention (e.g., prescription for someone with heart disease to a community walking group)	Healthcare settings outside of primary care (i.e., secondary, tertiary, inpatient)
Phenomenon of Interest	The prescription or use of NDIs (including dietary, physical, psychological, and self-management interventions with non-drug components) for the prevention, treatment, or management of a chronic condition/s (e.g., cardiovascular, cancer, diabetes, musculoskeletal, chronic respiratory, mental health, neurological)	Only use of pharmacological or surgical interventions; interventions to manage acute conditions
Design	Reviews of primary studies with systematic methods (e.g., systematic or scoping reviews) that include relevant evaluation outcomes collected by any means (e.g., questionnaire, focus group, interviews etc).	Rapid reviews, non-systematic literature reviews, overviews of systematic reviews, primary studies, study protocols, editorials, commentaries, abstracts
Evaluation	Barriers and/or enablers reported by clinicians, patients, or health systems to using or adhering to NDIs.	Barriers and enablers not reported, or reported combined with pharmacological/surgical interventions. Reviews reporting ‘associated factors’ (e.g., clinical or sociodemographic factors associated with exercise).
Research Type	Any review type meets other criteria if majority of data was primarily qualitative or mixed methods.	Syntheses that included quantitative data only.

^a^Primary care clinicians include health professionals that provide care to patients in primary healthcare settings, such as general practice, primary care, and community clinics. Examples include general practitioners/family physicians, primary care nurses, dietitians, physiotherapists, exercise physiologists, etc. [27]

### Search

#### Information sources

We searched 5 electronic databases: PubMed (MEDLINE), Cochrane Database of Systematic Reviews, EMBASE, PsycInfo, and CINAHL from inception to 7 September 2022 without language restrictions.

#### Search strategy

The search strategy combined free text and MeSH terms around 4 key concepts: ‘non-drug interventions’, ‘barriers/enablers’, ‘primary care’ and ‘systematic/scoping review’, and was peer-reviewed by a librarian, with additional input from a senior information specialist (Additional File 2). The search string was developed for PubMed and was translated using the Polyglot Search translator [28]. We deduplicated search results using an automated duplicate detection tool [25].

### Study selection

**Fig. 1:**
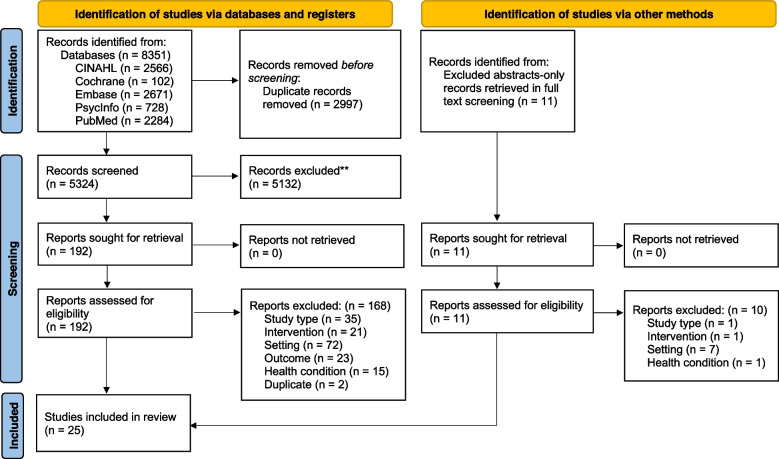
PRISMA 2020 Study 1 Flow Diagram

Two reviewers (HG and AD) independently screened deduplicated titles and abstracts against inclusion criteria (Table 1). Non-English title and abstracts were translated using Google translate, though no eligible non-English studies were identified. Two reviewers (HG and AD) independently screened full text articles (retrieved by HG). Any disagreements were resolved by discussion or referral to another reviewer (LA), if no consensus. We manually searched for and screened full text articles of abstract-only records excluded at the full text stage (“other methods”). A flow diagram is used to represent study selection (Fig. 1) [29].

### Quality assessment

#### Risk of bias assessment

We adapted AMSTAR 2 risk of bias (RoB) assessment tool referring to items from the CASP tool for systematic reviews, to assess RoB in reviews of qualitative studies. This adapted AMSTAR2 was piloted on two reviews by two independent reviewers (HG and LA) with good agreement (see Additional File 3 for a summary of changes). One reviewer (LA) assessed the RoB of the remaining included reviews. We elected to use an adapted AMSTAR tool as no dedicated tool to assess reviews of qualitative studies exists and our study includes a combination of qualitative, quantitative and mixed methods studies. The adjustments made were informed by examining JBI systematic review tools which includes some guidance on assessing quality of qualitative reviews [30]. We also extracted the RoB of the primary studies in included reviews. We did not independently assess the RoB of primary studies, if not reported by the original review authors.

#### Certainty of evidence

When reported in the included systematic reviews, we extracted the GRADE-CERQual assessment conducted by the original review author. We did not independently assess the certainty of evidence if not reported.

### Data collection and analysis

One reviewer (HG) extracted data from all review articles using a data extraction sheet piloted by two reviewers (HG and AD) on 10% of included reviews. A second independent reviewer (AD) validated extracted data and coding for 10% of reviews and was discussed between the two reviewers (HG and AD) until agreement achieved. If multiple reviews of the same NDI and condition (e.g., exercise for heart disease) were found, the most recent review was extracted first, working backward by publication date until no new barriers or enablers were identified. In instances where data was unclear or incomplete, study authors were contacted. The following data was extracted from reviews:

- Characteristics: countries of included primary studies, number and type of included primary studies, number of participants, participant type and description, reported chronic condition, reported NDI, method used for analysis, method used for RoB assessment;
- Primary study overlap: Included primary studies within reviews and RoB rating;
- Outcomes: barriers and/or enablers including theme and certainty of evidence.

Two key determinant frameworks (i.e., frameworks which help understand or explain factors which influence implementation outcomes) were applied in this review of reviews. First, the Consolidated Framework of Implementation Research (CFIR) was used to assess contextual factors to inform implementation strategies [31]. Reflective of the dynamic nature of implementation theory, CFIR was recently updated to centre intervention recipients and include equity determinants, with further critique and advancements encouraged by the authors [31]. Second, the Theoretical Domains Framework (TDF), which was developed to understand health professional implementation behaviour and is used to examine influences on behaviour [20].

We deductively coded each extracted barrier or enabler extracted using CFIR (and TDF if in the Individual CFIR domain). We examined each CFIR and TDF code to identify barrier and enabler theme and subtheme groups. During this, CFIR and TDF codes were iteratively revised to ensure consistency across extracted barriers and enablers. Due to the variety of interventions identified, barriers and enablers were not tabulated by intervention or condition, as per protocol.

We assessed the degree of overlap between primary studies by building a matrix of included reviews and their included primary studies. We used the graphical representation of the degree of overlap (GROOVE) tool to graphically represent the degree of overlap and calculate the Corrected Covered Area (CCA), a measure of the degree of overlap between primary studies [32–34]. Overlap is considered slight if the CCA is < 5%, moderate if it is ≥5% and < 10%, high if it is ≥10% and < 15%, and very high if CCA is ≥15% [33, 34]. Due to the variation in primary study health condition and NDI, structural missingness was not assessed.

## Results

### Study selection

After deduplication, 5324 title and abstract records were screened to identify 192 full-text records of which we included 24 reviews. One study was identified via other methods (Fig. 1). Overall, 25 reviews were included (Fig. 1). See Supplementary Table 1, Additional File 4 for a list of excluded reviews and reasons for exclusion.

### Characteristics of included reviews

Characteristics of included reviews (and the primary studies included in those reviews, where relevant) are summarised in Table 2; see Supplementary Table 2, Additional File 5 for detailed characteristics. Primary studies of included reviews were conducted in 24 countries across Africa, Asia, Europe, the Americas, and Oceania, but mostly in Europe or North America. Most included reviews reported data from patients (*n* = 23, 92%) and included qualitative (*n* = 24, 96%) or mixed methods (*n* = 8, 32%) primary studies. Included reviews were conducted across a range of specific chronic health conditions, while some reviews included studies from various chronic health conditions (*n* = 5, 20%). Physical activity or exercise were the most common NDIs examined in included reviews (*n* = 10, 40%). Some reviews addressed several interventions (e.g., included a combination of physical and nutritional components; *n* = 4, 16%).

**Table 2:** Summary of key characteristics of included reviews (*n* = 25) and their primary studies (*n* = 452)

Characteristics of included reviews (*n* = 25)	N (%)
Types of participants	
Patients	23 (92%)
Clinicians	9 (36%)
Family members/caregivers	4 (16%)
Other^a^	5 (20%)
Data types in included reviews	
Qualitative	24 (96%)
Quantitative	8 (32%)
Mixed Methods	13 (52%)
Not reported	1 (4%)
Health condition type	
Cardiovascular	4 (16%)
Diabetes	6 (24%)
Mental Health	2 (8%)
Musculoskeletal	4 (16%)
Neurological	2 (8%)
Renal	1 (4%)
Respiratory	1 (4%)
Mixed chronic conditions	5 (20%)
Intervention type	
Nutrition	2 (8%)
Physical	10 (40%)
Psychological	3 (12%)
Combined	4 (16%)
Self-management	6 (24%)
Characteristics of primary studies (n = 452)	Count (%)
Participants	44,852
Patients	36,367 (81%)
Clinicians	8154 (18%)
Family members/caregivers	66 (0.1%)
Other^a^	95 (0.2%)
Count by type not specified	270 (1%)
Not reported	3 studies
Regions	24 countries
Africa	1 (0.2%)
Asia	19 (4%)
Europe	185 (41%)
North America	129 (29%)
South America	3 (1%)
Oceania	43 (10%)
Not reported	4 (1%)

^a^Other participant types include coaches, teachers, school staff, community leaders and healthcare administrators

### Primary study overlap

Twenty-five included reviews had 452 unique primary studies. Of these, 410 appear in 1 review, 15 appear in 2 reviews, and 4 appear in 3 reviews. There is slight (< 5%) overlap of primary studies between included reviews (CCA = 0.21%). Of 300 nodes (pairs of review), 296 nodes have slight overlap (< 5%), while 4 nodes have very high (≥15%) overlap (Additional File 6). We identified two of these review pairs ([35, 36] and [37, 38]) during data extraction. As per protocol, we extracted the most recently published review first, and only extracted novel barriers and enablers for the older review. Another review [39] had very high overlap with 2 reviews [37, 38], but was not identified during data extraction, so extracted it in full. As no measure of effect size is estimated, we took no further action.

### Critical appraisal of included reviews

#### Included reviews

**Fig. 2:**
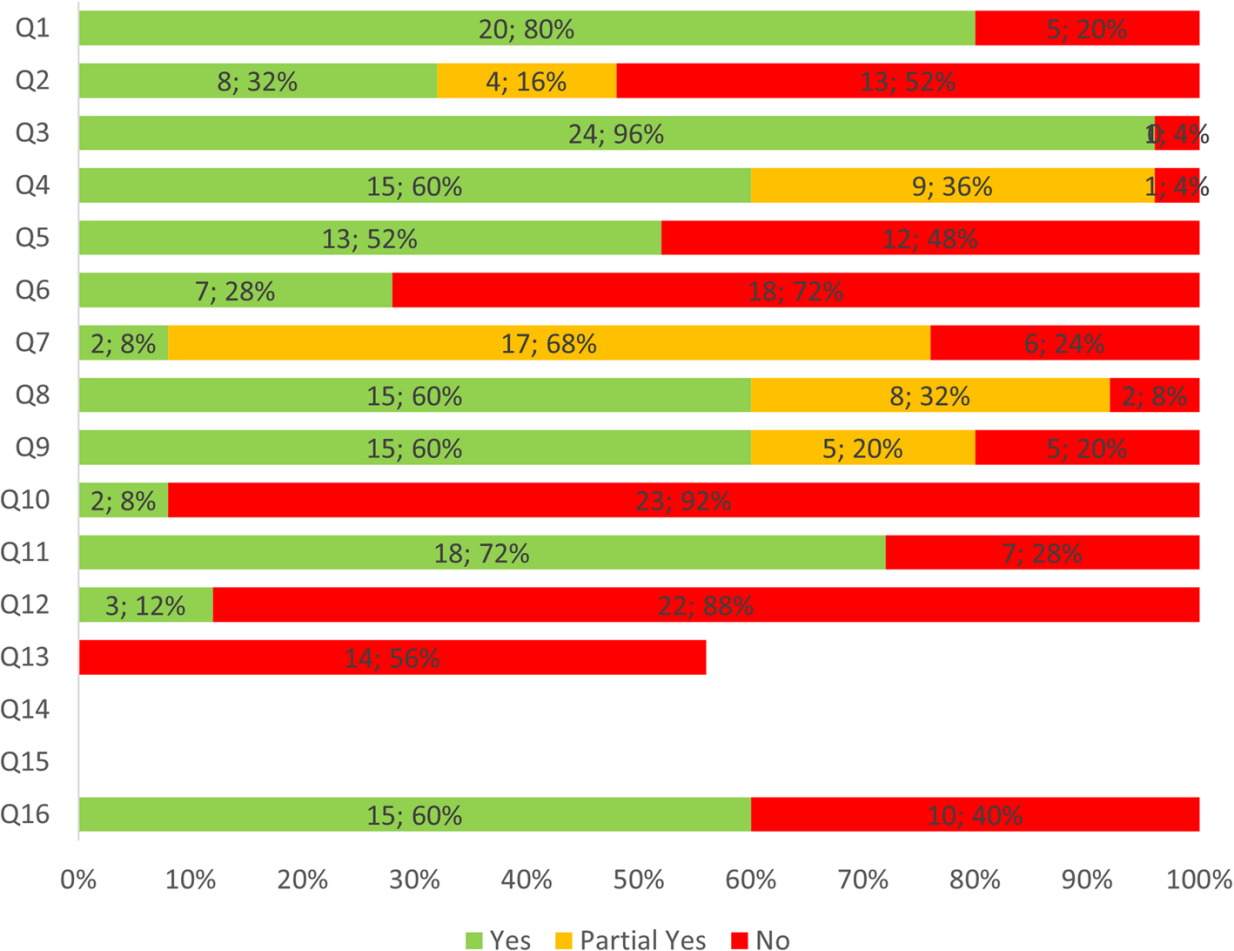
Modified AMSTAR rating by question

**Fig. 3:**
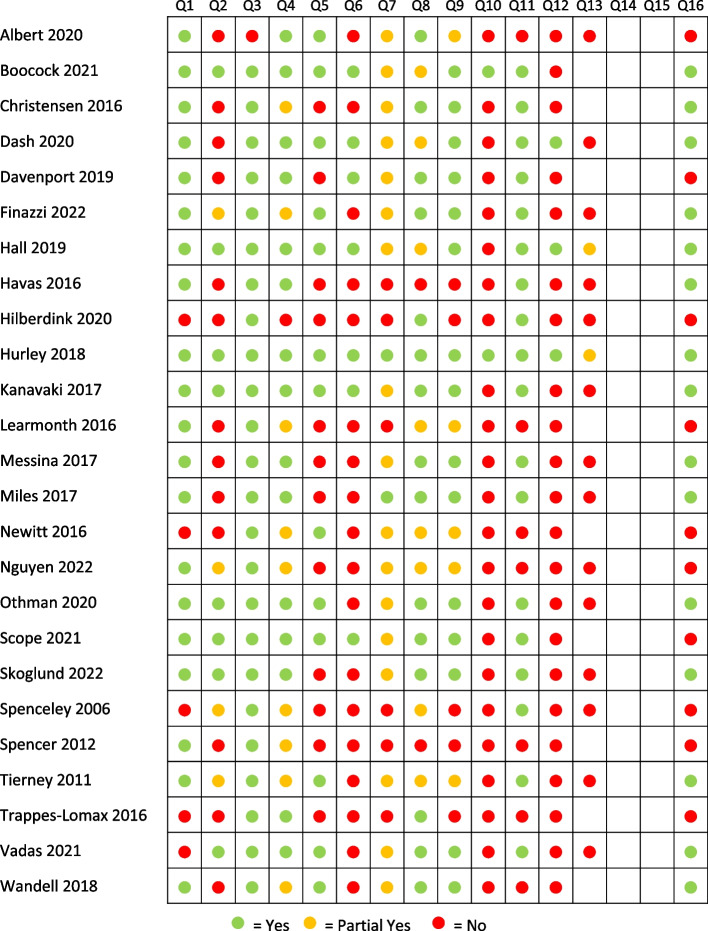
Modified AMSTAR rating by study

Modified AMSTAR assessment indicates most reviews used a comprehensive search strategy (*n* = 15, Yes 60%; *n* = 9, Partial Yes 36%), a satisfactory technique for assessing RoB (*n* = 15, Yes 60%; *n* = 5, Partial Yes 20%) and appropriate methods to combine results (*n* = 18; Yes 72%). Approximately half the reviews had a registered protocol (*n* = 8, Yes 32%; *n* = 4, Partial Yes 16%). In most reviews, authors did not: perform data extraction in duplicate (*n* = 18, No 72%), report sources of primary study funding (*n* = 23, No 92%), or assess the potential impact of primary study RoB on the results (*n* = 22, No 88%). AMSTAR ratings are represented graphically by question (Fig. 2) and by study (Fig. 3). See Additional file 7 for a summary of primary study RoB.

### Summary of findings

We identified 71 barrier and 59 enabler subthemes across 4 CFIR domains: Innovation, Outer Setting, Inner Setting, and Individuals [31]. See Table 3 for subthemes for Innovation, Inner Setting and Outer Setting domains, and Table 4 for subthemes related to Individual Domain with TDF codes [20].

**Table 3:** Barriers and enablers for innovation, inner setting, and outer setting CFIR domains

CFIR Domain	CFIR Construct	Theme	Barrier Subtheme	Enabler Subtheme
Innovation *STs: 7B and 5E*	Innovation relative advantage	*Relative benefit of NDI in health management* *STs: 2B and 1E*	^a^Relative ease of medication (HCP) [18] ^a^Don’t see role of NDI as relevant to health management (HCP, P) [40–44]	Clear utility of NDI (HCP) [18]
	Innovation adaptability	*Flexibility of intervention characteristics and delivery* *STs: 1B and 1E*	^a^Generalised or “one size fits all” interventions perceived as impersonal or patronising (HCP, P) [37, 38, 40, 45]	^a^Flexible, tailored or personalised interventions facilitate acceptability of and engagement in intervention (HCP, P) [35, 37, 38, 40–43, 45–51]
	Innovation complexity	*NDI content is complex* *STs: 2B*	Intervention contains mixed messages or counterintuitive information (P, HCP) [35, 37, 38, 44] Intervention difficult to apply for patients with multiple conditions (HCP) [43]	NR
	Innovation design	*Intervention delivery logistics* *STs: 1B and 3E*	Characteristics of group intervention not acceptable (P, HCP) [45, 50]	Remote or online delivery is acceptable, cost effective, convenient and reduces stigma (P, HCP) [40, 42, 46, 50] Group intervention in community setting (P, HCP) [37–39, 47, 50, 52] Monitoring during intervention supported accountability (P) [46, 52]
	Innovation cost	*Costs associated with intervention* *ST: 1B*	^a^Actual or perceived cost associated with intervention (P) [37, 38, 40, 46, 52–55]	NR
Outer setting *STs: 9B and 7E*	Local attitudes	*Attitudes and awareness towards medical condition in local community* *STs: 2B and 1E*	^a^Negative societal attitudes or pressures (P) [18, 38, 39, 43, 45, 54, 55] Perception that support is only available for some individuals (P) [37]	Societal awareness of medical condition (P) [38]
	Local conditions	*Access to facilities and services* *STs: 4B and 2E*	^a^Lack of available goods or services to access NDI (P) [36, 38, 44, 46, 53] Difficulties with transport (e.g., long distances to access suitable facilities) (P) [38] Weather and climate affect access (P) [36, 38, 46, 49, 52] Language or cultural barriers (P, HCP, HS) [41, 45, 52, 55]	^a^Ease of access to spaces and places (e.g., activities, green space) to participate in NDI (P) [46, 56] Public transport available to access NDI (P) [38]
	Policies and Laws	*Guidelines and legislation* *STs: 1B and 2E*	Attitudes towards, awareness of, and complexity of guidelines (P, HCP) [40, 41, 57]	Guidelines are useful to inform views about NDI (HCP) [40, 44] Legislation against harmful health behaviour (P) [41]
	Financing	*Authority support for NDI programs* *ST: 1E*	NR	Financial assistance and support from authorities for NDI programs (P) [41, 46]
	External pressure	*Sociocultural pressures* *STs: 2B*	^a^Sociocultural pressure related to diet, culture or gender roles (P) [18, 46, 53, 55, 58] Medicolegal concerns (HCP) [43]	
	Societal pressure	*Information in media* *ST: 1E*	NR	Information in media about condition or NDI (P) [41, 45]
Inner setting *STs: 20B and 21E*	Structural characteristics	*see below subconstructs*	*see below subconstructs*	*see below subconstructs*
	Physical infrastructure	*Physical accessibility of facilities* *ST: 1B*	Accessibility of facilities, including parking [NDI facility] (P) [37–39]	
	IT infrastructure	*Availability of software to adequately support NDI delivery* *ST: 2B and 1E*	Lack of IT support or suitable software [health service] (HCP) [41] Issues when things go wrong with technology [health service] (P) [42]	Availability and integration of software, including software support, patient records, appointments [health service] (HCP) [41]
	Work infrastructure	*Availability of staff to provide NDI support* *STs: 2B and 2E*	Lack of clarity around staffing levels and qualifications [health service] (HS) [18, 41] Poor leadership (HCP) [41]	Role of nurses to support NDI activities [health service] (HCP) [18, 40, 41, 52, 58] Availability of female GPs [health service] (HCP) [41]
	Relational connections	*Personal relationships* *STs: 2B and 2E* *Therapeutic relationships* *STs: 1B and 2E* *Professional relationships* *STs: 1B and 1E*	^a^Lack of social support from family or friends [home environment] (P) [35–41, 49, 51, 54, 55, 57] ^a^Lack of connection to others with the same health condition [community] (P) [38, 50] ^a^Poor relationship between patient and HCP due to care provision, disagreement or lack of trust [health service] (P, HCP) [18, 37, 40–43, 51, 55–58] Poor or fragmented connections between different healthcare providers, including referral pathways [health service] (HCP, HS) [18, 41, 51]	^a^Support and encouragement from family and friends [home environment] (P) [37–40, 42, 46–49, 54–58] ^a^Engagement with and support from others with the same condition [community] (P) [37–40, 43, 45–47, 49–51, 56–58] ^a^High quality relationship between patient and clinician [health service] (P, HCP) [18, 37–43, 45, 47, 54, 55] External support from advocacy groups or support worker [community] (P) [40] Good relationships between healthcare providers [health service] (HCP, HS) [41]
	Communication	*Communication between HCP and patient* *STs: 2B and 4E*	Poor or ineffective communication between patient and HCP [health service] (P, HCP) [39–41, 51, 58] Difficulty accessing information on patients [health service] (HCP) [41]	Ongoing communication between patient and HCP [health service[(P, HCP) [37, 38, 42] Respectful, empowering communication from HCP to patients [health service] (P) [40, 46, 50, 52] Opportunity to talk about experience in consultation [health service] (P) [50] Timely, clear and simple communication about NDI [health service] (P, HCP) [18, 38, 46, 48]
	Culture	*Primary care as a key setting for NDIs* *STs: 1B and 1E*	Primary care seen as not responsible to for providing NDI [health service] (HS) [41, 52]	Primary care is a key appropriate setting for prevention activities [health service] (HS) [41, 43, 52]
	Recipient-Centredness	*HCPs attitudes towards patient* *STs: 1B and 1E*	Poor or discriminatory HCP attitude of towards patient [health service] (P) [18, 43, 44, 48, 52, 55]	Non-judgemental HCP attitude towards patient [health service] (P) [18]
	Compatibility	*Opportunity to conduct NDI activity in consultation* *STs: 1B and 1E*	NDI not acceptable to clinicians due to impracticality/time [health service] (HCP) [40, 41]	Option to do NDI in context of health checks and prevention activities [health service] (HS) [41]
	Incentive systems	*Incentivisation* *ST: 1E*	NR	Provide incentives to motivate participation [health service] (P) [56]
	Available resources	*Access to NDI prescription* *STs: 2B*	^a^Insufficient time or staff to adequately provide NDI intervention prescription [health service] (HCP) [18, 37, 38, 40, 41, 43, 44, 48, 51, 52, 54, 56, 58] ^a^Difficulty accessing NDI prescription service due to availability or appointment times [health service] (P) [38, 40, 41, 44, 45, 52, 55]	
	Space	*Space available* *STs: 1B and 1E*	Lack of space to complete NDI [NDI facility] (HS) [41]	Availability of space to complete NDI [NDI facility] (P) [37, 38]
	Materials and equipment	*Education material available* *STs: 1B and 1E* *Facilities available* *STs: 1B and 1E*	^a^Lack of patient education materials [health service] (P) [18, 39, 41] Facilities in NDI environment do not accommodate for needs associated with health condition (e.g., wait times, lack of resting places or hand holds) [NDI facility] [37–39]	^a^Relevant patient education materials [health service] (P) [40, 45, 47, 51, 57] Safe equipment and environment to complete NDI [NDI facility] (P) [37–39]
	Access to knowledge and information	*Training opportunities for staff* *STs: 1B and 1E* *Access to education material* *STs: 1E*	Lack of time for or availability of training opportunities for staff about NDI [health service] (HCP) [18, 40]	Sufficient clinician training in NDI [health service] (HCP) [18, 43, 52, 56] ^a^Patients have access to reliable education regarding health condition and NDI [health service] (P) [40, 46, 47, 50, 52, 54, 55]

*NDI* Non-drug intervention, *ST/s* Subtheme/s, *B* Barrier, *E* Enabler, *NR* None reported, ^a^key subtheme discussed in text, *HCP* barrier or enabler for clinician, *P* barrier or enabler for patient (or their carer), *HS* barrier or enabler for healthcare system

**Table 4:** Barriers and enablers for individual CFIR domain with TDF

CFIR Construct	TDF Domain	Theme	Barrier Subtheme	Enabler Subtheme
Capability *STs: 9B and 5E*	Knowledge	*Knowledge and awareness* *STs: 3B and 1E*	^a^Lack of knowledge or awareness of condition and/or management of condition (P, HCP) [35, 36, 39–41, 43, 45, 52–54] ^a^Lack of knowledge about NDIs or conservative management (P, HCP) [18, 36, 38, 42, 44, 45, 52, 53, 55] Low general health literacy (P) [51, 58]	^a^Knowledge about the condition and/or condition management (P) [36, 43, 47, 54, 55, 57]
	Skills	*Health condition management skills* *STs: 3B and 3E*	^a^Lack of skills to clearly and effectively communicate about benefits, harms, or lifestyle changes (HCP) [40, 41, 43, 44, 53–55] ^a^Lack of skills to effectively self-manage condition (P) [38, 40, 48, 54, 55] Lack of experience in managing condition with NDI (HCP) [41, 43]	^a^Skills to effectively self-manage condition (P) [38, 40, 43, 46, 47, 52, 57] Good general coping skills (P) [47, 54, 55] Clinician skills to support patient, including motivation and monitoring (HCP) [41, 45, 47]
	Memory, attention and decision processes	*Memory and decision making* *STs: 2B*	Forget to engage in NDI (P, HCP) [38, 44] Energy limitations due to health condition impacts decision between life activities and NDI (P) [37, 42]	
	Behavioural regulation	*Routine and adherence* *STs: 1B and 1E*	Difficulties adhering to condition management program that uses NDI (P) [41, 46, 57, 58]	Day to day routine that enables and prioritises condition management with NDI (P) [38–40, 47, 55, 57]
Opportunity *STs: 8B and 6E*	Environmental context and resources	*Personal circumstances and resources* *STs: 6B and 4E*	^a^Symptom of condition (e.g., pain, fatigue) interfere with engagement in NDI (P) [35–39, 42, 43, 47, 49, 51, 52, 55, 56, 58] ^a^Other comorbidity interfere with engagement in NDI (P) [38, 40, 49, 50, 52, 54, 55] Personal financial disadvantage (P) [38, 39, 51, 58] ^a^Personal and family stressors interfere with engagement in NDI (P) [37, 38, 40, 46, 47, 52, 53, 55] Current lifestyle interferes with engagement in NDI (P) [52] High demands of NDI limit engagement (P) [47, 55]	Ability to engage in NDI at home (P) [37, 47] Disease condition is stable (P) [47] Time available to engage in NDI (P) [47] Having a pet (P) [39, 49]
	Social influences	*Influence of others and community* *STs: 2B and 2E*	Concerns about being a burden (P) [40, 42] Comparison of self to others (P) [35, 55]	People facilitating NDI in the community were understanding and encouraging (P) [36–38] Engagement with religion supported emotional changes (P) [58]
Motivation *STs: 18B and 15E*	Social or professional roles and identity	*Roles and identities* *STs: 1B and 3E*	Loss of identity or capacity as a result of health condition (P) [35, 36, 38, 39, 55]	HCPs perceive delivery of NDI to be a part of their role, and act on this (HCP) [18, 39, 52] Engagement with NDI doesn’t interfere with ‘normal’ function, sense of self or identity (P) [36, 39, 46, 57] NDI becomes part of sense of self or identity (P) [46]
	Beliefs about capabilities	*Confidence and self-efficacy* *STs: 3B and 2E*	Low self-efficacy and confidence to manage condition (P, HCP) [38, 40, 43, 46, 52, 57] Lack of confidence to use tools associated with NDI (P, HCP) [40] Overconfidence in current knowledge about NDI (P) [18]	Confidence to engage in discussion about NDI with patients (HCP) [18, 43] Self-efficacy and confidence to self-manage condition with NDI (P) [40, 42, 47, 57]
	Optimism	*Outlook on health condition* *STs: 1B and 1E*	Negative attitude towards NDI (P) [35, 38]	Attitude of positivity and optimism towards NDI and health condition management (P) [35, 36, 39, 48, 49, 57]
	Intentions	*Intention to engage in NDI* *STs: 1B and 1E*	Diagnosis of health condition triggers engagement in NDI (P) [39]	Uncommitted or unwilling to engage in NDI (P) [38, 40]
	Goals	*Goal setting and motivation* *STs: 1B and 2E*	Absence of goals around self-management/NDI (P) [40, 48]	Making plans and setting goals around condition management and NDI (P) [35, 36, 39, 40, 46, 47, 49, 56] Motivated to engage in NDI (P) [39, 41, 47]
	Beliefs about consequences	*Beliefs and assumptions* *STs: 5B and 1E*	^a^Belief that using a NDI is useless or harmful (P, HCP) [35–38, 40, 42, 43, 51, 52] Assumptions about patient motivation deters clinician from offering NDI (HCP) [52] ^a^Disbelief or denial about diagnosis inhibited engagement in NDI (P) [38, 40, 46, 53] Making lifestyle changes perceived as too challenging (P) [46, 52] Preference for alternative medicine (P) [40, 58]	^a^NDI viewed as way to control condition and condition progression [35, 36, 39, 40]
	Reinforcement	*Reinforcement and feedback loops* *STs: 1B and 3E*	Self-monitoring tools onerous to maintain (P) [37, 38]	^a^Reminder/monitoring systems for progress, medication and symptoms are motivating (P) [37, 38, 40, 46, 52] ^a^Health improvements seen as result of engagement with NDI, prompting further engagement (P) [36–39, 42, 46, 47, 49, 55] Prior positive experience with NDI (P) [37, 38, 45, 46]
	Emotion	*Emotions as a regulator of engagement* *STs: 5B and 2E*	Negative emotion (e.g., despondency, despair) associated with delivering the intervention (HCP) [18, 42, 43] ^a^Negative emotion (e.g., fear, anxiety, powerlessness) inhibit engagement with NDI (P) [18, 38, 40, 46–50] ^a^Negative emotion (e.g., depression, anxiety) impact general wellbeing, coping and self-esteem (P) [38–40, 42, 49, 51, 56] Frustration with reduced capacity/independence that comes with health condition (P) [38, 49] Negative emotion (e.g., scepticism, apathy, disappointment) towards NDI (P) [38, 45]	Feeling positive and safe when engaging in NDI (P) [37–39] ^a^Negative emotion (e.g., guilt, shame) motivated engagement with NDI (P) [40, 46, 47, 52]

*NDI* Non-drug intervention, *ST/s* Subtheme/s, *B* Barrier, *E* Enabler, *NR* None reported, ^a^key subtheme discussed in text, *HCP* barrier or enabler for clinician, *P* barrier or enabler for patient (or their carer), *HS* barrier or enabler for healthcare system

#### Innovation domain

Across constructs of *Innovation Relative Advantage, Innovation Adaptability, Innovation Complexity, Innovation Design* and *Innovation Cost,* we identified seven barrier and five enabler subthemes from 21 reviews [31] (Table 3), and highlight five key subthemes (4 barriers and 1 enabler).

##### Flexibility of intervention characteristics and delivery (CFIR: innovation adaptability)

We identified two factors around the theme of intervention flexibility. When the intervention adopts a ‘one size fits all’ approach, patients and clinicians perceive it as impersonal or patronising, creating a barrier (4 reviews [37, 38, 40, 45]). *“A lot of the participants reported feeling they already knew the information presented in [e-cognitive behavioural therapy], and that this was not tailored to their individual needs and situation […] there was a general sense that it was not a complete intervention, often summed up in a phrase to the effect that ‘It wasn’t for me but could help someone else’”* [45]. Conversely, a flexible, tailored or personalised intervention facilitates both patient and clinician acceptability of and engagement with the NDI (14 reviews [35, 37, 38, 40–43, 45–51], low certainty of evidence from 1 review [43]).

##### Costs associated with the intervention (CFIR: innovation cost)

We identified that actual or perceived costs of the intervention as a key barrier for patients (8 reviews [37, 38, 40, 46, 52–55]). *“Look, the barrier to those goal settings is budget, you know […] So, don’t go telling poor people ‘you’re going to get diabetes if you eat this and this and this’; so we want you to eat this food, but it’s too expensive for you to buy, you know”* [46]*.*

##### Relative benefit of NDIs (CFIR: innovation relative advantage)

One review reported a barrier that healthcare practitioners were less likely to prescribe a NDI due to comparative ease of prescribing medication (moderate to high certainty of evidence from 1 review [18]). This may relate to clinician or patient perception that NDIs are irrelevant to health management (5 reviews [40–44], 2 reviews reported variable certainty of evidence: very low [44], high [43]).

#### Outer setting domain

We identified nine barriers and seven enabler subthemes from 19 reviews across constructs of *Local Attitudes, Local Conditions, Policies and Laws, Financing, External Pressure,* and subconstruct *Societal Pressure* [31] (Table 3) and highlight four key subthemes (three barriers and 1 enabler).

##### Attitudes and awareness towards medical condition in local community (CFIR: local attitudes)

We identified negative societal attitudes towards the health condition as a barrier for patients in seven reviews [18, 38, 39, 43, 45, 54, 55] (low certainty evidence from one review [18]). Examples included peer pressure to fit in by concealing health condition symptoms and social stigma (e.g., actual or perceived negative perception towards health condition). *“Most participants described negative experiences of being misunderstood, judged and stigmatised because of their depression. They learned to mask their feelings, adopting an appearance of wellness and keeping people at a distance. Most participants described this as an isolating experience”* [45].

##### Sociocultural pressures (CFIR: external pressure)

Similarly, we identified external sociocultural pressures (e.g., the intersection between food and culture or traditional gender roles around food preparation), as a patient barrier in five reviews [18, 46, 53, 55, 58] (low certainty evidence from 1 study [18]). *“My whole family eats white rice since young, it has become a habit, a culture in us. Now say change to brown rice, not easy, it takes time for us to adjust to the new taste of brown rice”* [46]*.*

##### Access to facilities and services (CFIR: local condition)

Access to facilities and services is an important factor for patients. Five reviews [36, 38, 44, 46, 53] identified lack of available goods or services to access the NDI as a barrier (moderate to high certainty evidence from 2 reviews [36, 44]). Ease of access to places/spaces to participate in the NDI enabled engagement (2 reviews [46, 56]).

#### Inner setting domain

We identified 20 barrier and 21 enabler subthemes from 24 reviews across constructs of *Structural Characteristics [all subconstructs], Relational Connections, Communication, Culture [Recipient Centredness subconstruct], Compatibility, Incentive Systems, Available Resources [Space, and Materials and Equipment subconstructs],* and *Access to Knowledge and Information)* [31] (Table 3). Eleven key subthemes (six barriers and five enablers) are highlighted.

##### Personal, therapeutic, and professional relationships (CFIR: relational connections)

The therapeutic relationship between clinician and patient is crucial. When poor, due to lack of continuity, disagreement or distrust, it can act as a barrier for both parties (11 reviews [18, 37, 40–43, 51, 55–58]). Conversely, a high-quality, trusting relationship enables use of NDIs (12 reviews [18, 37–43, 45, 47, 54, 55]). Similarly, lack of support from family/friends or others with the same condition acts as a barrier (12 reviews [35–41, 49–51, 54, 55, 57]), while good relationships, support and encouragement serve as an enabler (18 reviews [37–40, 42, 43, 45–51, 54–58]). *“And I found the whole process valuable, particularly going along with other people who had similar problems and sharing their problems with them”* [56]*.* Three reviews reported variable certainty of evidence ratings for these themes: low [43], moderate [18, 43], high [18, 36, 43].

##### Access to NDI prescriptions and information (CFIR: available resources, access to knowledge and information)

Patients and clinicians face barriers in accessing NDI prescriptions and information about them. A patient barrier in 6 reviews was the challenge in accessing services for NDI prescription, due to service unavailability or prohibitive wait times [4–6, 8, 18, 20, 24]. Insufficient time or staffing to provide the NDI prescription was a key barrier for clinicians in health service settings (13 reviews [18, 37, 38, 40, 41, 43, 44, 48, 51, 52, 54, 56, 58]). Other important patient enablers were the availability of, and access to, relevant education materials (availability: 5 reviews [40, 45, 47, 51, 57], high certainty evidence from one review [18]) (access: seven reviews [40, 46, 47, 50, 52, 54, 55]). Lack of these materials is a barrier (three reviews [18, 39, 41]).

#### Individual domain

Across the roles subdomain constructs of *Innovation Deliverers, Innovation Recipients,* and *Other Implementation Support* and characteristics subdomain constructs of *Capability, Opportunity* and *Motivation,* we identified 35 barrier and 26 enabler subthemes from 25 reviews, which mapped to all TDF domains [20] (Table 4). Seventeen key subthemes (11 barriers and 6 enablers) are highlighted.

##### Knowledge and awareness (CFIR: capability; TDF: knowledge)

We identified lack of knowledge about the health condition (10 reviews [35, 36, 39–41, 43, 45, 52–54], high certainty evidence from 1 review [43]) or NDI (9 reviews [18, 36, 38, 42, 44, 45, 52, 53, 55], variable certainty evidence from 3 reviews [18, 36, 44]) as a major patient and clinician barrier. *“Most [general practitioners] were unfamiliar with the conservative interventions other than medication, such as cognitive-behavioural therapy, spinal manipulations, and exercises.”* [44]. Conversely, knowledge about the health condition or health condition management is a patient enabler in 6 reviews [36, 43, 47, 54, 55, 57], with moderate certainty evidence from 1 review [43].

##### Health condition management skills (CFIR: capability; TDF: skills)

Clinicians face a key barrier in lack of skills to clearly and effectively communicate about benefits and harms or lifestyle changes (seven reviews [40, 41, 43, 44, 53–55], moderate to high certainty evidence from two reviews [43, 44]). For patients, lack of self-management skills acts as a barrier (five reviews [38, 40, 48, 54, 55]), while presence of self-management skills enables use of NDIs (seven reviews [38, 40, 43, 46, 47, 52, 57], moderate certainty evidence from 1 review [43]).

##### Personal circumstances and resources (CFIR: opportunity; TDF: environmental context and resources)

Patient’s symptoms such as pain or fatigue, due to the target health condition (14 reviews [35–39, 42, 43, 47, 49, 51, 52, 55, 56, 58], high certainty evidence from 1 review [36]) or a comorbid health condition (7 reviews [38, 40, 49, 50, 52, 54, 55]), inhibit NDI engagement. *“I can’t exercise too much sometimes. My knees can’t take it. Because we are getting on in years as well as sometimes it hurts. There was once when I went for a walk and I had leg cramps after I went back. It’s really painful”* [58]. Barriers of personal or family stressors, such as workload or difficulties accessing childcare, also interfere with NDI engagement (8 reviews [37, 38, 40, 46, 47, 52, 53, 55]). *“I am always so busy*. *.. in the evenings there are always papers to look at, I have no time for exercise.. . I simply don’t have the time”* [46].

##### Beliefs and assumptions (CFIR: motivation; TDF: beliefs about consequences)

The belief that using a NDI is either useless or harmful is a barrier for clinicians and patients (9 reviews [35–38, 40, 42, 43, 51, 52], variable certainty evidence from 2 reviews: low [43]; high [36]). *“…there is nothing that can be done about the [osteoarthritis]; therefore, I do nothing…”* [36]. However, the perception that the NDI can control the condition and condition progression is an enabler (4 reviews [35, 36, 39, 40], low certainty evidence from 1 review [36]). Disbelief or denial about the health condition also inhibits NDI engagement for patients (4 reviews [38, 40, 46, 53]).

##### Reinforcement and feedback loops (CFIR: motivation, TDF: reinforcement)

Several factors enable NDI engagement via reinforcement. Patient health improvements from using the NDI prompts further engagement (9 reviews [36–39, 42, 46, 47, 49, 55], high certainty evidence from 1 review [36]). Using reminder or monitoring systems to track progress, medication or symptoms is also helpful (5 reviews [37, 38, 40, 46, 52] *““I have a Fitbit that makes it easier, because I like to challenge myself to make sure I get my steps every day. So, lots of times, I’ll get home in the evening and I’ll see them at 9000 steps, and I’ll like go out and walk up and down the driveway”* [46]*.*

##### Emotion as a regulator of engagement (CFIR: motivation, TDF: emotion)

Negative emotions regulate patient engagement with NDIs in various ways. Emotions like fear, anxiety, and powerlessness can inhibit engagement with NDIs (8 reviews [18, 38, 40, 46–50], moderate certainty evidence from 1 review [18]). Feelings of depression or anxiety can impact general wellbeing, coping and self-esteem (7 reviews [38–40, 42, 49, 51, 56]). Interestingly, emotions like guilt and shame can enable engagement with NDIs (4 reviews, [40, 46, 47, 52]).

## Discussion

In this overview of reviews, we aimed to thematically synthesise reviews examining barriers and enablers to using NDIs for chronic condition management in primary care. Overall, across 25 included reviews, we identified 71 common barrier and 59 common enabler subthemes across Innovation, Inner Setting, Outer Setting and Individual CFIR Domains [31]. As the included reviews examined barriers and enablers to using NDIs after implementation in practice, we did not identify any subthemes for the CFIR Process domain. We also examined factors identified in the Individual domain using the TDF to allow for more in-depth analysis [20, 59].

Key themes related to the Innovation (NDI) are flexible intervention characteristics for patients and clinicians alike, costs associated with the intervention for patients, and the relative benefit of NDIs. The ability to tailor or personalise an NDI facilitates engagement, while a “one size fits all” approach is perceived as impersonal and represents a barrier. Recent meta-ethnographic evidence suggests that patients receiving weight management care from their general practitioners sought care tailored to their individual needs. However, general practitioners may be ill-equipped to provide individualised advice, due to lack of available guidance, training or resources [60]. Providing clinicians with appropriate training and resources to tailor NDIs may enable clinicians to provide this personalised advice. Actual or perceived NDI costs (e.g., cost associated with a prescribed dietary strategy or exercise plan) are an engagement barrier for patients. Many studies examine cost-effectiveness of delivering NDIs from a health system perspective (e.g., nutrition care [61] or hypertension treatments [62]), but there is less consideration of the intervention cost to patients. There may be an incorrect perception that NDIs are costly. For example, while there are some programs for internet-delivered cognitive behavioural therapy for depression that have associated costs, there are also no-cost options available [63]. Ensuring that NDIs are affordable compared to drug interventions, supporting patients to access no or low-cost options, providing comparison of medication vs NDI costs, and challenging beliefs that they are inherently unaffordable, may help support uptake of NDIs. Although only identified in one included review, the barrier of clinician not prescribing NDIs due to the comparative ease of prescribing medication is noteworthy. Although some progress has been made on NDI prescription, particularly for exercise [64, 65], further examination of the NDI prescription process would be beneficial.

Some Outer Setting (e.g., community) factors influence patient engagement in NDIs, include local attitudes towards the health condition, sociocultural pressures, and access to facilities and services. The first two factors are difficult to address, but lack of available goods and services to access the NDI once prescribed is potentially changeable. Although not in primary care, recent systematic review evidence shows that a major external factor influencing self-management for chronic pain was intervention accessibility, including location of facilities and service availability [66]. Having accessible spaces and places to engage with prescribed NDIs, as well as having available community services to provide NDIs may enhance engagement with NDIs. For example, a water-based exercise prescription for knee osteoarthritis is inappropriate if the patient does not have access to private or public pool facilities.

We identified two key themes in the Inner Setting domain: access to NDIs (both prescription and information), and personal, therapeutic, and professional relationships. The availability of, and access to educational information about NDIs enables patient engagement, though the availability of these resources may be lacking. Online non-drug resources like Royal Australian College of General Practitioners’ Handbook of Non-Drug Interventions (HANDI) provides information for clinicians, though some interventions also have patient resources [67]. There is some evidence from a recent, unpublished Australian survey exploring clinicians awareness and use of HANDI, that one-third of clinicians are unaware of this resource, and of those that are aware, half rarely use it [68]. Given that this is a clinician-focussed resource, without clinician guidance it is unlikely that patients will be aware of such guidelines. Improving educational materials about NDIs, and enhancing access to these materials, may support delivery of NDIs in primary care. Further, for patients, accessing services to prescribe NDIs can be a challenge, possibly related to the clinician barrier of insufficient time or staffing to deliver the NDI. A systematic review of barriers and enablers to implementation of physical activity interventions in primary care found similar barriers in the Environmental Context and Resources TDF domain, including lack of professionals to deliver the intervention [69]. Similarly, evidence from a narrative review examining underuse of NDIs for headache found there are few clinicians trained in NDI approaches, potentially explaining these access difficulties [70]. The therapeutic relationship between clinician and patient can act as a barrier or enabler to use of NDIs, depending on the quality of the relationship. It has been well-established that quality of the relationship between clinicians and patients has a positive effect on patient outcomes [71, 72]. Our results suggest that an aligned clinician-patient relationship can influence engagement in NDIs. This is somewhat supported by recent integrative review evidence that person-centred communication and trust in the relationship was an important factor in lifestyle risk communication [73]. Several of the factors in this domain (access to educational resources and skills to communicate about NDIs) are related to findings in the Individual domain.

Within the Individual domain, some key, interrelated factors influence use of NDIs include knowledge and awareness, skills to manage the health condition with NDIs, and assumptions and beliefs about NDIs. Lack of knowledge about NDIs is a major barrier for both clinicians and patients, and has been consistently identified as a factor in previous research [66, 74]. For clinicians, this may be due to a lack of awareness of non-drug guidelines [75], and broader lack of training, skills or confidence in skills in delivering NDIs [73]. For patients, a problem with availability of, or access to, NDI educational resources may explain this knowledge gap. We also found that some patients and clinicians believe NDIs are useless or harmful. Improving availability of, or access to, high quality information about NDI may have the dual benefit of addressing this belief and improving knowledge about NDIs more broadly. Another major barrier for clinicians is a lack of skills to communicate about risk or lifestyle changes clearly and effectively, also related to other theme of confidence and self-efficacy. An integrative review of nurses delivery of lifestyle interventions found that nurses lacked the knowledge, skills, and confidence to deliver NDIs, providing support that these factors are interrelated [76].

Identification of common factors impacting use of non-drug interventions for chronic conditions in primary care has several possible applications. This includes as a starting-point for developing implementation strategies for specific non-drug interventions, identifying top-level implementation strategies for addressing multiple non-drug intervention simultaneously, or to inform important factors for scale-up of existing non-drug interventions. Existing tools, such as the CFIR-ERIC matching tool, can be used to map identified barrier and enabler factors to effective implementation strategies [77, 78]. For example, for key Inner Setting domain factor of availability of, and access to, patient education material about NDIs, implementation activities may include conducting educational meetings, accessing new funding, and developing and distributing education material [77].

This review has several strengths. First, this study goes beyond condition or intervention-specific barriers and enablers to identify common factors across NDIs. Second, we used the updated CFIR framework to analyse factors at Innovation, Outer Setting, Inner Setting and Individual domain levels [31] and applied the TDF to gain a more nuanced view of the Individual domain [20, 59]. Finally, we used rigorous methods: we developed a comprehensive search with a search specialist and librarian, two reviewers screened reviews, we pre-specified a study protocol [21] and reported the results in accordance with the PRIOR checklist [23]. There are several limitations that should be considered when interpreting the study findings. First, the data was extracted by one author (HG). Although a second author validated 10% of extracted data and codes, with good agreement (~ 90%), the review could have been improved by a second author coding all extracted data. Given the use of a coding framework (instead of iteratively developed codes) and that the data in included reviews was generally qualitative (i.e., no quantitative estimate of effect was measured). We determined our approach sufficient for the review type. Second, we did not conduct a forward and backward citation searching as specified in the protocol. Due to the breadth of included reviews and that we identified many common barriers and enablers, after data extraction we determined that inclusion of further reviews would not likely provide any additional barrier or enabler subthemes. Third, RoB assessment was conducted by one author (LA), with 10% validated by a second author (HG). As assessment of RoB is somewhat contentious for qualitative syntheses, due to the inherently subjective nature of the data [79], we determined this approach sufficient for completeness of reporting but recognise the limitation of our decision. This is also reflected in the RoB assessments of primary studies within included reviews. While most of these did conduct some form of RoB assessment, many did not provide an overall rating, though this is a limitation of the literature, not the study (Additional File 7). Fourth, as there is currently no dedicated tool for assessing RoB of qualitative reviews, we used the prominent systematic review appraisal tool, AMSTAR 2, and made adjustments to also assess qualitative reviews. This adjusted tool is not validated, representing a limitation. However, given that quality assessment of qualitative review is contentious [79], no overall ratings have been given, and the quality of reviews had no bearing on inclusion, we believe this adjusted tool is sufficient for the intended purpose (reporting of quality of included reviews). Validation of this tool or development of quality appraisal tool for qualitative reviews may be a future direction of research.

## Conclusions

As prevalence of chronic conditions is expected to rise [3], it is crucial to understand factors that help and hinder effective treatment, including non-drug treatments. This overview used implementation frameworks (CFIR and TDF) to synthesise 71 common barriers and 69 common enablers to using effective NDIs. Our findings can be used to inform top-level implementation strategies or scale-up of the adoption of NDIs across various conditions and settings. By understanding common factors affecting the use of NDIs generally, broader, and generalisable implementation interventions can be developed to address multiple NDIs.

### Supplementary Information

**Supplementary Material 1.**

**Supplementary Material 2.**

**Supplementary Material 3.**

**Supplementary Material 4.**

**Supplementary Material 5.**

**Supplementary Material 6.**

**Supplementary Material 7.**

**Supplementary Material 8.**

## Data Availability

All data generated or analysed during this study are included in this published article as a supplementary information file (see Additional File 8).

## Abbreviations

CCA: Corrected Covered Area
CFIR: Consolidated Framework of Implementation Research
GROOVE: Graphical representation of the degree of overlap
HANDI: Handbook of Non-Drug Interventions
NDI(s): Non-drug intervention(s)
NHANES: National Health and Nutrition Examination Survey
PRIOR: Preferred Reporting Items for Overview of Reviews
RoB: Risk of Bias
SPIDER: Sample, Phenomenon of Interest, Design, Evaluation, Research type
TDF: Theoretical Domains Framework
US: United States
