# Supplementary material for: Common barriers and enablers to the use of non-drug interventions for managing common chronic conditions in primary care: an overview of reviews
Source: BMC Prim Care. 2024 Apr 6;25:108. doi: 10.1186/s12875-024-02321-8 (PMC10998330; doi:10.1186/s12875-024-02321-8)
Supplement: Supplementary file 2 — Supplementary Material 2. [file 12875_2024_2321_MOESM2_ESM.docx]

Additional File 2: Search strategy

### PubMed (MEDLINE)

(non-pharmaceutical[tiab] OR non-pharmacological[tiab] OR nonpharmaceutical[tiab] OR nonpharmacological[tiab] OR non-drug[tiab] OR nondrug[tiab] OR self-management[tiab] OR self-practice[tiab] OR advice[tiab] OR behaviour[tiab] OR diet[ti] OR dietary[ti] OR nutrition[ti] OR exercise[ti] OR physical[ti] OR psychotherapy[ti] OR lifestyle[ti] OR prevention[ti] OR “self-management”[MeSH Terms] OR “diet”[MeSH Terms] OR “exercise”[MeSH Terms] OR “physical therapy modalities”[MeSH Terms] OR “psychotherapy”[MeSH Terms] OR “healthy lifestyle”[MeSH Terms])

AND

(barrier[tiab] OR barriers[tiab] OR enable[tiab] OR enabler[tiab] OR enablers[tiab] OR facilitate[tiab] OR facilitators[tiab] OR strategy[tiab] OR strategies[tiab] OR themes[tiab])

AND

(“primary care”[tiab] OR practitioners[tiab] OR physicians[tiab] OR “healthcare professionals”[tiab] OR “health care professionals”[tiab] OR “health professionals”[tiab] OR “Primary health”[tiab] OR “Primary care”[tiab] OR “General practice”[tiab] OR “General practices”[tiab] OR “General practitioners”[tiab] OR “General practitioner”[tiab] OR “Family practice”[tiab] OR “Family practitioners”[tiab] OR “Family practitioner”[tiab] OR “Family physician”[tiab] OR “Family physicians”[tiab] OR “Primary Health Care”[MeSH Terms])

AND

("Meta analysis"[pt] OR "Systematic review"[pt] OR ((Search[tiab] OR Searched[tiab]) AND (PubMed[tiab] OR MEDLINE[tiab])) OR ((Systematic[tiab] OR Scoping[tiab]) AND (Review[tiab])) OR "Meta analysis"[tiab] OR Meta-analysis[tiab] OR Review[ti] OR ((Systematically[tiab] OR Reviewed[tiab]) AND (literature[tiab])) OR "Cochrane Database Syst Rev"[jour])

### Cochrane Database of Systematic Review

(non-pharmaceutical:ti,ab OR non-pharmacological:ti,ab OR nonpharmaceutical:ti,ab OR nonpharmacological:ti,ab OR non-drug:ti,ab OR nondrug:ti,ab OR self-management:ti,ab OR self-practice:ti,ab OR advice:ti,ab OR behaviour:ti,ab OR diet:ti OR dietary:ti OR nutrition:ti OR exercise:ti OR physical:ti OR psychotherapy:ti OR lifestyle:ti OR prevention:ti OR [mh “self-management”] OR [mh diet] OR [mh exercise] OR [mh "physical therapy modalities"] OR [mh psychotherapy] OR [mh "healthy lifestyle"])

AND

(barrier:ti,ab OR barriers:ti,ab OR enable:ti,ab OR enabler:ti,ab OR enablers:ti,ab OR facilitate:ti,ab OR facilitators:ti,ab OR strategy:ti,ab OR strategies:ti,ab OR themes:ti,ab)

AND

("primary care":ti,ab OR practitioners:ti,ab OR physicians:ti,ab OR "healthcare professionals":ti,ab OR "‘health care professionals’":ti,ab OR "health professionals":ti,ab OR "Primary health":ti,ab OR "Primary care":ti,ab OR "General practice":ti,ab OR "General practices":ti,ab OR "General practitioners":ti,ab OR "General practitioner":ti,ab OR "Family practice":ti,ab OR "Family practitioners":ti,ab OR "Family practitioner":ti,ab OR "Family physician":ti,ab OR "Family physicians":ti,ab OR [mh "Primary Health Care"])

AND

("Meta analysis":pt OR "Systematic review":pt OR ((Search:ti,ab OR Searched:ti,ab) AND (PubMed:ti,ab OR MEDLINE:ti,ab)) OR ((Systematic:ti,ab OR Scoping:ti,ab) AND (Review:ti,ab)) OR "Meta analysis":ti,ab OR Meta-analysis:ti,ab OR Review:ti OR ((Systematically:ti,ab OR Reviewed:ti,ab) AND (literature:ti,ab)) OR "Cochrane Database Syst Rev[jour]")

### EMBASE

(non-pharmaceutical:ti,ab OR non-pharmacological:ti,ab OR nonpharmaceutical:ti,ab OR nonpharmacological:ti,ab OR non-drug:ti,ab OR nondrug:ti,ab OR self-management:ti,ab OR self-practice:ti,ab OR advice:ti,ab OR behaviour:ti,ab OR diet:ti OR dietary:ti OR nutrition:ti OR exercise:ti OR physical:ti OR psychotherapy:ti OR lifestyle:ti OR prevention:ti OR ‘self-care’/exp/mj OR nutrition/exp/mj OR ‘physical activity’/exp/mj OR physiotherapy/exp/mj OR psychotherapy/exp/mj OR 'healthy lifestyle'/exp/mj)

AND

(barrier:ti,ab OR barriers:ti,ab OR enable:ti,ab OR enabler:ti,ab OR enablers:ti,ab OR facilitate:ti,ab OR facilitators:ti,ab OR strategy:ti,ab OR strategies:ti,ab OR themes:ti,ab)

AND

('primary care':ti,ab OR practitioners:ti,ab OR physicians:ti,ab OR 'healthcare professionals':ti,ab OR 'health care professionals':ti,ab OR 'health professionals':ti,ab OR 'Primary health':ti,ab OR 'Primary care':ti,ab OR 'General practice':ti,ab OR 'General practices':ti,ab OR 'General practitioners':ti,ab OR 'General practitioner':ti,ab OR 'Family practice':ti,ab OR 'Family practitioners':ti,ab OR 'Family practitioner':ti,ab OR 'Family physician':ti,ab OR 'Family physicians':ti,ab OR 'Primary Health Care'/exp/mj)

AND

([cochrane review]/lim OR [systematic review]/lim OR [meta analysis]/lim OR ((Search:ti,ab OR Searched:ti,ab) AND (PubMed:ti,ab OR MEDLINE:ti,ab)) OR (Systematic:ti,ab AND Review:ti,ab) OR 'Meta analysis':ti,ab OR Meta-analysis:ti,ab OR Review:ti OR ((Systematically:ti,ab OR Reviewed:ti,ab) AND (literature:ti,ab)))

**Note:** article type term was replaced with a specialised Embase term designed by search specialist; subject heading terms limited to major

### CINAHL

((TI non-pharmaceutical OR AB non-pharmaceutical) OR (TI non-pharmacological OR AB non-pharmacological) OR (TI nonpharmaceutical OR AB nonpharmaceutical) OR (TI nonpharmacological OR AB nonpharmacological) OR (TI non-drug OR AB non-drug) OR (TI nondrug OR AB nondrug) OR (TI self-management OR AB self-management) OR (TI self-practice OR AB self-practice) OR (TI advice OR AB advice) OR (TI behaviour OR AB behaviour) OR TI diet OR TI dietary OR TI nutrition OR TI exercise OR TI physical OR TI psychotherapy OR TI lifestyle OR TI prevention OR (MH "Self Care+") OR (MH "Diet+") OR (MH "Exercise+") OR (MH "Physical Therapy+") OR (MH "Psychotherapy+") OR (MH "Life Style+"))

AND

((TI barrier OR AB barrier) OR (TI barriers OR AB barriers) OR (TI enable OR AB enable) OR (TI enabler OR AB enabler) OR (TI enablers OR AB enablers) OR (TI facilitate OR AB facilitate) OR (TI facilitators OR AB facilitators) OR (TI strategy OR AB strategy) OR (TI strategies OR AB strategies) OR (TI themes OR AB themes))

AND

((TI "primary care" OR AB "primary care") OR (TI practitioners OR AB practitioners) OR (TI physicians OR AB physicians) OR (TI "healthcare professionals" OR AB "healthcare professionals") OR (TI "‘health care professionals’" OR AB "‘health care professionals’") OR (TI "health professionals" OR AB "health professionals") OR (TI "Primary health" OR AB "Primary health") OR (TI "Primary care" OR AB "Primary care") OR (TI "General practice" OR AB "General practice") OR (TI "General practices" OR AB "General practices") OR (TI "General practitioners" OR AB "General practitioners") OR (TI "General practitioner" OR AB "General practitioner") OR (TI "Family practice" OR AB "Family practice") OR (TI "Family practitioners" OR AB "Family practitioners") OR (TI "Family practitioner" OR AB "Family practitioner") OR (TI "Family physician" OR AB "Family physician") OR (TI "Family physicians" OR AB "Family physicians") OR (MH "Primary Health Care"))

AND

(PT "Meta analysis" OR PT "Systematic review" OR (((TI Search OR AB Search) OR (TI Searched OR AB Searched)) AND ((TI PubMed OR AB PubMed) OR (TI MEDLINE OR AB MEDLINE))) OR (((TI Systematic OR AB Systematic) OR (TI Scoping OR AB Scoping)) AND ((TI Review OR AB Review))) OR (TI "Meta analysis" OR AB "Meta analysis") OR (TI Meta-analysis OR AB Meta-analysis) OR TI Review OR (((TI Systematically OR AB Systematically) OR (TI Reviewed OR AB Reviewed)) AND ((TI literature OR AB literature))) OR "Cochrane Database Syst Rev[jour]")

### PsycInfo

(non-pharmaceutical.ti,ab. OR non-pharmacological.ti,ab. OR nonpharmaceutical.ti,ab. OR nonpharmacological.ti,ab. OR non-drug.ti,ab. OR nondrug.ti,ab. OR self-management.ti,ab. OR self-practice.ti,ab. OR advice.ti,ab. OR behaviour.ti,ab. OR diet.ti. OR dietary.ti. OR nutrition.ti. OR exercise.ti. OR physical.ti. OR psychotherapy.ti. OR lifestyle.ti. OR prevention.ti. OR exp Self-Management/ OR exp Diets/ OR exp Exercise/ OR exp Physical Therapy/ OR exp Psychotherapy/ OR exp Lifestyle/)

AND

(barrier.ti,ab. OR barriers.ti,ab. OR enable.ti,ab. OR enabler.ti,ab. OR enablers.ti,ab. OR facilitate.ti,ab. OR facilitators.ti,ab. OR strategy.ti,ab. OR strategies.ti,ab. OR themes.ti,ab.)

AND

("primary care".ti,ab. OR practitioners.ti,ab. OR physicians.ti,ab. OR "healthcare professionals".ti,ab. OR "health care professionals".ti,ab. OR "health professionals".ti,ab. OR "Primary health".ti,ab. OR "Primary care".ti,ab. OR "General practice".ti,ab. OR "General practices".ti,ab. OR "General practitioners".ti,ab. OR "General practitioner".ti,ab. OR "Family practice".ti,ab. OR "Family practitioners".ti,ab. OR "Family practitioner".ti,ab. OR "Family physician".ti,ab. OR "Family physicians".ti,ab. OR exp Primary Health Care/)

AND

("Meta analysis".md. OR "Systematic review".md. OR ((Search.ti,ab. OR Searched.ti,ab.) AND (PubMed.ti,ab. OR MEDLINE.ti,ab.)) OR ((Systematic.ti,ab. OR Scoping.ti,ab.) AND (Review.ti,ab.)) OR "Meta analysis".ti,ab. OR Meta-analysis.ti,ab. OR Review.ti. OR ((Systematically.ti,ab. OR Reviewed.ti,ab.) AND (literature.ti,ab.)))

***Note:*** removed Cochrane term, changed .pt. publication type to .md methodology

### MeSH terms translated into subject headings for different databases

| **MESH for PubMed, Cochrane** | **Emtree for Embase** | **CINAHL Subject Headings For CINAHL** | **For PsycInfo** |
| --- | --- | --- | --- |
| self-management | 'self care'/exp | (MH "Self Care+") | exp Self-Management/ |
| diet | 'nutrition'/exp | (MH "Diet+") | exp Diets/ |
| exercise | ‘physical activity’/exp | (MH "Exercise+") | exp Exercise/ |
| physical therapy modalities | ‘physiotherapy’/exp | (MH "Physical Therapy+") | exp Physical Therapy/ |
| psychotherapy | 'psychotherapy'/exp | (MH "Psychotherapy+") | exp Psychotherapy/ |
| healthy lifestyle | 'healthy lifestyle'/exp | (MH "Life Style+") | exp Lifestyle/ |
| Primary Health Care | 'primary health care'/exp | (MH "Primary Health Care") | exp Primary Health Care/ |
