# Supplementary material for: Common barriers and enablers to the use of non-drug interventions for managing common chronic conditions in primary care: an overview of reviews
Source: BMC Prim Care. 2024 Apr 6;25:108. doi: 10.1186/s12875-024-02321-8 (PMC10998330; doi:10.1186/s12875-024-02321-8)
Supplement: Supplementary file 3 — Supplementary Material 3. [file 12875_2024_2321_MOESM3_ESM.docx]

Additional File 3: Modified AMSTAR Questions

***Note:*** *As we could not find an appropriate RoB tool for qualitative systematic reviews, we revised the AMSTAR 2 tool to accommodate for the included qualitative systematic reviews in our study. Changes from the original AMSTAR 2 tool are highlighted in green. Additionally, no overall rating was given.*

**Q1. Did the research questions and inclusion criteria for the review include the components of PICO?**

1. Yes
2. No

*For* ***Yes****, all PICO elements must be present. For qualitative: SPIDER criteria is also acceptable*

- Population (or sample)
- Intervention (or phenomenon of interest)
- Outcomes
- Optional: Comparator group
- Optional: timeframe for follow up

**Q2. Did the report of the review contain an explicit statement that the review methods were established prior to the conduct of the review and did the report justify any significant deviations from the protocol?**

1. Yes
2. Partial Yes
3. No

*For* ***Partial Yes:*** *The authors state that they had a written protocol or guide that included ALL the* following:

- review question(s) or aims
- a search strategy
- inclusion/exclusion criteria
- a risk of bias assessment

*For* ***Yes:*** *partial yes plus the protocol should be registered and should also have specified:*

- protocol is registered
- a meta-analysis/synthesis plan, if appropriate
- justification for any deviations from the protocol
- Optional: a plan for investigating causes of heterogeneity

**Q3. Did the review authors explain their selection of the study designs for inclusion in the review?**

1. Yes
2. No

*For* ***Yes****, the review should satisfy ONE of the following:*

- Explanation for including only RCTs
- OR Explanation for including only NRSI
- OR Explanation for including only Qualitative
- OR Explanation for including combination of study types

**Q4. Did the review authors use a comprehensive literature search strategy?**

1. Yes
2. Partial Yes
3. No

*For* ***Partial Yes:*** *(all the following):*

- searched at least 2 databases (relevant to research question)
- provided key word and/or search strategy
- justified publication restrictions (e.g. language)

*For* ***Yes:*** *partial yes plus should also have (all the following):*

- searched the reference lists / bibliographies of included studies
- included/consulted content experts in the field (including in authorship list)
- conducted search within 24 months of completion of the review
- Optional: searched for grey literature
- Optional: searched trial/study registries

**Q5. Did the review authors perform study selection in duplicate?**

1. Yes
2. No

*For* ***Yes****, (either ONE of the following):*

- at least two reviewers independently agreed on selection of eligible studies AND achieved consensus on which studies to include
- OR two reviewers selected a sample of eligible studies AND achieved good agreement (at least 80 percent), with the remainder selected by one reviewer.

**Q6. Did the review authors perform data extraction in duplicate?**

1. Yes
2. No

*For* ***Yes****, (either ONE of the following):*

- at least two reviewers achieved consensus on which data to extract from included studies
- OR two reviewers extracted data from a sample of eligible studies AND achieved good agreement (at least 80 percent), with the remainder extracted by one reviewer
- OR justification for why data extraction was not completed in duplicate

**Q7. Did the review authors provide a list of excluded studies and justify the exclusions?**

1. Yes
2. Partial Yes
3. No

*For* ***Partial Yes:***

- Recorded the number of excluded studies with reasons for exclusion (e.g., in a PRISMA diagram)

*For* ***Yes:*** *partial yes PLUS must also have:*

- Provided a list of all potentially relevant studies that were read in full-text form but excluded from the review
- AND justified the exclusion from the review of each potentially relevant study

**Q8. Did the review authors describe the included studies in adequate detail?**

1. Yes
2. Partial Yes
3. No

*For* ***Partial Yes:*** *(ALL the following)*

- described populations
- described interventions
- described outcomes
- described research designs
- Optional: described comparators

*For* ***Yes:*** *partial yes PLUS should also have ALL the following:*

- described population in detail
- described intervention in detail
- described study’s setting
- Optional: described comparator in detail
- Optional: timeframe for follow-up

**Q9RCT. Did the review authors use a satisfactory technique for assessing the risk of bias in individual studies that were included in the review?**

1. Yes
2. Partial Yes
3. No

*For* ***Partial Yes:*** *(must have assessed RoB from both)*

- unconcealed allocation
- lack of blinding of patients and assessors when assessing outcomes (unnecessary for objective outcomes such as all cause mortality)

*For* ***Yes:*** *partial yes PLUS must also have assessed RoB from both*

- allocation sequence that was not truly random
- selection of the reported result from among multiple measurements or analyses of a specified outcome

**Q9NRSI. Did the review authors use a satisfactory technique for assessing the risk of bias in individual studies that were included in the review?**

1. Yes
2. Partial Yes
3. No

*For* ***Partial Yes:*** *(must have assessed RoB from both)*

- confounding
- selection bias

*For* ***Yes:*** *partial yes PLUS must also have assessed RoB from both*

- methods used to ascertain exposures and outcomes
- selection of the reported result from among multiple measurements OR analyses of a specified outcome

**Q9Qual. Did the review authors use a satisfactory technique for assessing the risk of bias in individual studies that were included in the review?**

1. Yes
2. Partial Yes
3. No

*For* ***Partial Yes:*** *(must have assessed RoB from at least two the following sources)*

*For* ***Yes:*** *(must have assessed RoB from ALL the following sources)*

- Appropriateness of qualitative approach
- Congruency between stated methods and synthesis results
- Quality of the methodology, data collection and analysis
- The role of the researcher

**Q10. Did the review authors report on the sources of funding for the studies included in the review?**

*Note: Reporting that the reviewers looked for this information but it was not reported by study authors also qualifies*

1. Yes
2. No

*For* ***Yes****, must have*

- reported on the sources of funding for individual studies included in the review

**Q11RCT. *If meta-analysis was performed*, did the review authors use appropriate methods for statistical combination of results?**

1. Yes
2. No
3. No meta-analysis conducted

*For* ***Yes****, (all must be selected):*

- The authors justified combining the data in a meta-analysis
- They used an appropriate weighted technique to combine study results and adjusted for heterogeneity if present
- Investigated the causes of any heterogeneity

**Q11NRSI. *If meta-analysis was performed,* did the review authors use appropriate methods for statistical combination of results?**

1. Yes
2. No
3. No meta-analysis conducted

*For* ***Yes****, (all must be selected):*

- The authors justified combining the data in a meta-analysis
- they used an appropriate weighted technique to combine study results, adjusting for heterogeneity if present
- they statistically combined effect estimates from NRSI that were adjusted for confounding, rather than combining raw data, OR justified combining raw data when adjusted effect estimates were not available
- they reported separate summary estimates for RCTs and NRSI separately when both were included in the review

**Q11Qual. *If meta-synthesis was performed,* did the review authors use appropriate methods for combination of results?**

1. Yes
2. No
3. No meta-synthesis was conducted

*For* ***Yes****, (all must be selected):*

- The authors used an appropriate qualitative evidence synthesis technique to combine their results (e.g., meta-ethnography, thematic analysis, grounded theory etc)
- methods that have been used to synthesize findings congruent with the stated methodology of the review
- adequate descriptive and explanatory information to support the final synthesized findings that have been constructed from the findings sourced from the original research

**Q12. *If meta-analysis or synthesis was performed,* did the review authors assess the potential impact of RoB in individual studies on the results of the meta-analysis or other evidence synthesis?**

1. Yes
2. No
3. No meta analysis or synthesis was conducted

*For* ***Yes****, (ONE must be selected):*

- included only low risk of bias RCTs
- OR, if the pooled estimate was based on RCTs and/or NRSI at variable RoB, the authors performed analyses to investigate possible impact of RoB on summary estimates of effect.
- OR if a synthesis of qualitative studies at variable RoB, the authors provided justification for why they did or did not investigate the impact of RoB on synthesis

**Q13. Did the review authors account for RoB in individual studies when interpreting/discussing the results of the review?**

1. Yes
2. No
3. N/A (qualitative)

*For* ***Yes****, (ONE must be selected):*

- included only low risk of bias RCTs
- OR, if RCTs with moderate or high RoB, or NRSI were included the review provided a discussion of the likely impact of RoB on the results

**Q14. Did the review authors provide a satisfactory explanation for, and discussion of, any heterogeneity observed in the results of the review?**

1. Yes
2. No
3. N/A (qualitative)

*For* ***Yes****, (ONE must be selected):*

- There was no significant heterogeneity in the results
- OR if heterogeneity was present the authors performed an investigation of sources of any heterogeneity in the results and discussed the impact of this on the results of the review

**Q15. *If they performed quantitative synthesis*, did the review authors carry out an adequate investigation of publication bias (small study bias) and discuss its likely impact on the results of the review?**

1. Yes
2. No
3. No meta-analysis conducted
4. N/A (qualitative)

*For* ***Yes****, (must be selected):*

- performed graphical or statistical tests for publication bias and discussed the likelihood and magnitude of impact of publication bias

**Q16. Did the review authors report any potential sources of conflict of interest, including any funding they received for conducting the review?**

1. Yes
2. No

*For* ***Yes****, (ONE must be selected):*

- The authors reported no competing interests
- OR the authors described their funding sources and how they managed potential conflicts of interest
