# Supplementary material for: Common barriers and enablers to the use of non-drug interventions for managing common chronic conditions in primary care: an overview of reviews
Source: BMC Prim Care. 2024 Apr 6;25:108. doi: 10.1186/s12875-024-02321-8 (PMC10998330; doi:10.1186/s12875-024-02321-8)
Supplement: Supplementary file 4 — Supplementary Material 4. [file 12875_2024_2321_MOESM4_ESM.docx]

Additional File 4: Reasons for exclusion

**Supplementary Table 1: Full text excluded studies and reasons for exclusion**

| **No.** | **Full Reference** | **Reason for exclusion** |
| --- | --- | --- |
| ***Excluded articles from original search*** | | |
| 1 | Aapro M, Bossi P, Dasari A, Fallowfield L, Gascón P, Geller M, et al. Digital health for optimal supportive care in oncology: benefits, limits, and future perspectives. Support Care Cancer. 2020;28(10):4589-612. DOI:10.1007/s00520-020-05539-1 | 2. Intervention (not NDI) |
| 2 | Abaraogu UO, Abaraogu OD, Dall PM, Tew G, Stuart W, Brittenden J, et al. Exercise therapy in routine management of peripheral arterial disease and intermittent claudication: a scoping review. Ther Adv Cardiovasc Dis. 2020;14:1753944720924270. DOI:10.1177/1753944720924270 | 3. Setting (not PC) |
| 3 | Abdin S, Heath G, Welch RK. Health professionals' views and experiences of discussing weight with children and their families: A systematic review of qualitative research. Child Care Health Dev. 2021;47(4):562-74. DOI:10.1111/cch.12854 | 3. Setting (not PC) |
| 4 | Abu-Janb N, Jaana M. Facilitators and barriers to adherence to gluten-free diet among adults with celiac disease: a systematic review. J Hum Nutr Diet. 2020;33(6):786-810. DOI:10.1111/jhn.12754 | 4. Outcome (not B+F) |
| 5 | Alaslawi H, Berrou I, Alhamid A, Alhuwail D, Aslanpour Z. Factors affecting the adoption of mobile apps for self-management of type 2 diabetes: A systematic review. International Journal of Pharmacy Practice. 2019;27:47. DOI:10.1111/ijpp.12533 | 1. Study type (not SR) |
| 6 | Albert FA, Crowe MJ, Malau-Aduli AEO, Malau-Aduli BS. Physical Activity Promotion: A Systematic Review of The Perceptions of Healthcare Professionals. Int J Environ Res Public Health. 2020;17(12). DOI:10.3390/ijerph17124358 | 3. Setting (not PC) |
| 7 | Alderman G, Semple S, Cesnik R, Toohey K. Health Care Professionals' Knowledge and Attitudes Toward Physical Activity in Cancer Patients: A Systematic Review. Semin Oncol Nurs. 2020;36(5):151070. DOI:10.1016/j.soncn.2020.151070 | 3. Setting (not PC) |
| 8 | Allison RL. Back to Basics: The Effect of Healthy Diet and Exercise on Chronic Disease Management. South Dakota medicine : the journal of the South Dakota State Medical Association. 2017:10-8. | 1. Study type (not SR) |
| 9 | Anderson EA, Armer JM. Factors Impacting Management of Breast Cancer-Related Lymphedema (BCRL) in Hispanic/Latina Breast Cancer Survivors: A Literature Review. Hisp Health Care Int. 2021;19(3):190-202. DOI:10.1177/1540415321990621 | 4. Outcome (not B+F) |
| 10 | Arnold A, Rosenthal M, Salkar M, Patelb S, Harrell E, Aldridge H, et al. Patient Centered Studies Focused on Type 2 Diabetes Management, Education, and Family Support: A Scoping Review. Curr Diabetes Rev. 2022;18(7):e171121197989. DOI:10.2174/1573399818666211117113026 | 4. Outcome (not B+F) |
| 11 | Arora A, Poudel P, Manohar N, Bhole S, Baur LA. The role of oral health care professionals in preventing and managing obesity: A systematic review of current practices and perceived barriers. Obes Res Clin Pract. 2019;13(3):217-25. DOI:10.1016/j.orcp.2019.03.005 | 5. Condition (not chronic) |
| 12 | Atkins L, Stefanidou C, Chadborn T, Thompson K, Michie S, Lorencatto F. Influences on NHS Health Check behaviours: a systematic review. BMC Public Health. 2020;20(1):1359. DOI:10.1186/s12889-020-09365-2 | 5. Condition (not chronic) |
| 13 | Avery L, Charman SJ, Taylor L, Flynn D, Mosely K, Speight J, et al. Systematic development of a theory-informed multifaceted behavioural intervention to increase physical activity of adults with type 2 diabetes in routine primary care: Movement as Medicine for Type 2 Diabetes. Implement Sci. 2016;11:99. DOI:10.1186/s13012-016-0459-6 | 4. Outcome (not B+F) |
| 14 | Avery L, Taylor L, Lievesley M, Mosely K, Speight J, Sniehotta FF, et al. Development of a behavioural intervention targeting free-living physical activity in adults with Type 2 diabetes in primary care: Movement as Medicine. Diabetic Medicine. 2012;29:100-1. DOI:10.1111/j.1464-5491.2011.03555_2.x | 1. Study type (not SR) |
| 15 | Baird LG, Fraser K. Home Care Case Managers' Integrated Care of Older Adults With Multiple Chronic Conditions: A Scoping Review. Prof Case Manag. 2018;23(4):165-89. DOI:10.1097/ncm.0000000000000286 | 2. Intervention (not NDI) |
| 16 | Ball L, Crowley J, Hiddink GJ. Nutrition care by general practitioners: An integrative review using the COM-B framework. Australian Journal of Primary Health. 2019;25(3):vi. DOI:10.1071/PYv25n3abs | 1. Study type (not SR) |
| 17 | Balogun-Katung A, Carswell C, Brown JVE, Coventry P, Ajjan R, Alderson S, et al. Exploring the facilitators, barriers, and strategies for self-management in adults living with severe mental illness, with and without long-term conditions: A qualitative evidence synthesis. PLoS One. 2021;16(10):e0258937. DOI:10.1371/journal.pone.0258937 | 3. Setting (not PC) |
| 18 | Band R, Bradbury K, Morton K, May C, Michie S, Mair FS, et al. Intervention planning for a digital intervention for self-management of hypertension: a theory-, evidence- and person-based approach. Implement Sci. 2017;12(1):25. DOI:10.1186/s13012-017-0553-4 | 1. Study type (not SR) |
| 19 | Beaudin J, Chouinard M-C, Girard A, Houle J, Ellefsen É, Hudon C. Integrated self-management support provided by primary care nurses to persons with chronic diseases and common mental disorders: a scoping review. BMC Nursing. 2022;21(1):1-19. DOI:10.1186/s12912-022-01000-2 | 4. Outcome (not B+F) |
| 20 | Bekele H, Asefa A, Getachew B, Belete AM. Barriers and Strategies to Lifestyle and Dietary Pattern Interventions for Prevention and Management of TYPE-2 Diabetes in Africa, Systematic Review. J Diabetes Res. 2020;2020:7948712. DOI:10.1155/2020/7948712 | 3. Setting (not PC) |
| 21 | Bhattarai P, Newton-John TRO, Phillips JL. Quality and Usability of Arthritic Pain Self-Management Apps for Older Adults: A Systematic Review. Pain Med. 2018;19(3):471-84. DOI:10.1093/pm/pnx090 | 4. Outcome (not B+F) |
| 22 | Birken SA, Ellis SD, Walker JS, DiMartino LD, Check DK, Gerstel AA, et al. Guidelines for the use of survivorship care plans: a systematic quality appraisal using the AGREE II instrument. Implement Sci. 2015;10:63. DOI:10.1186/s13012-015-0254-9 | 2. Intervention (not NDI) |
| 23 | Blasco-Blasco M, Puig-García M, Piay N, Lumbreras B, Hernández-Aguado I, Parker LA. Barriers and facilitators to successful management of type 2 diabetes mellitus in Latin America and the Caribbean: A systematic review. PLoS One. 2020;15(9):e0237542. DOI:10.1371/journal.pone.0237542 | 2. Intervention (not NDI) |
| 24 | Bouma SE, van Beek JFE, Diercks RL, van der Woude LHV, Stevens M, van den Akker-Scheek I. Barriers and facilitators perceived by healthcare professionals for implementing lifestyle interventions in patients with osteoarthritis: a scoping review. BMJ Open. 2022;12(2):e056831. DOI:10.1136/bmjopen-2021-056831 | 3. Setting (not PC) |
| 25 | Bradbury D, Chisholm A, Watson PM, Bundy C, Bradbury N, Birtwistle S. Barriers and facilitators to health care professionals discussing child weight with parents: A meta-synthesis of qualitative studies. British Journal of Health Psychology. 2018;23(3):701-22. DOI:10.1111/bjhp.12312 | 3. Setting (not PC) |
| 26 | Brebach R, Sharpe L, Costa DS, Rhodes P, Butow P. Psychological intervention targeting distress for cancer patients: a meta-analytic study investigating uptake and adherence. Psychooncology. 2016;25(8):882-90. DOI:10.1002/pon.4099 | 4. Outcome (not B+F) |
| 27 | Browall M, Mijwel S, Rundqvist H, Wengström Y. Physical Activity During and After Adjuvant Treatment for Breast Cancer: An Integrative Review of Women's Experiences. Integr Cancer Ther. 2018;17(1):16-30. DOI:10.1177/1534735416683807 | 3. Setting (not PC) |
| 28 | Buckley BS, Byrne MC, Smith SM. Service organisation for the secondary prevention of ischaemic heart disease in primary care. Cochrane Database Syst Rev. 2010(3):Cd006772. DOI:10.1002/14651858.CD006772.pub2 | 4. Outcome (not B+F) |
| 29 | Byron C, Savage E, Lehane E, Burton A, Cornally N. Self-management interventions to support help-seeking behaviours in inflammatory bowel disease: a scoping review. Gastrointestinal Nursing. 2022;20(6):30-41. DOI:10.12968/gasn.2022.20.6.30 | 2. Intervention (not NDI) |
| 30 | Campbell F, Lawton J, Rankin D, Clowes M, Coates E, Heller S, et al. Follow-Up Support for Effective type 1 Diabetes self-management (The FUSED Model): A systematic review and meta-ethnography of the barriers, facilitators and recommendations for sustaining self-management skills after attending a structured education programme. BMC Health Serv Res. 2018;18(1):898. DOI:10.1186/s12913-018-3655-z | 2. Intervention (not NDI) |
| 31 | Cardoso Barbosa H, de Queiroz Oliveira JA, Moreira da Costa J, de Melo Santos RP, Gonçalves Miranda L, de Carvalho Torres H, et al. Empowerment-oriented strategies to identify behavior change in patients with chronic diseases: An integrative review of the literature. Patient Educ Couns. 2021;104(4):689-702. DOI:10.1016/j.pec.2021.01.011 | 3. Setting (not PC) |
| 32 | Carter DD, Robinson K, Forbes J, Hayes S. Experiences of mobile health in promoting physical activity: A qualitative systematic review and meta-ethnography. PLoS One. 2018;13(12):e0208759. DOI:10.1371/journal.pone.0208759 | 3. Setting (not PC) |
| 33 | Chan PS, Fang Y, Wong MC, Huang J, Wang Z, Yeoh EK. Using Consolidated Framework for Implementation Research to investigate facilitators and barriers of implementing alcohol screening and brief intervention among primary care health professionals: a systematic review. Implement Sci. 2021;16(1):99. DOI:10.1186/s13012-021-01170-8 | 5. Condition (not chronic) |
| 34 | Clark AM, King-Shier KM, Duncan A, Spaling M, Stone JA, Jaglal S, et al. Factors influencing referral to cardiac rehabilitation and secondary prevention programs: a systematic review. Eur J Prev Cardiol. 2013;20(4):692-700. DOI:10.1177/2047487312447846 | 3. Setting (not PC) |
| 35 | Clark AM, King-Shier KM, Thompson DR, Spaling MA, Duncan AS, Stone JA, et al. A qualitative systematic review of influences on attendance at cardiac rehabilitation programs after referral. Am Heart J. 2012;164(6):835-45.e2. DOI:10.1016/j.ahj.2012.08.020 | 3. Setting (not PC) |
| 36 | Clarke AL, Jhamb M, Bennett PN. Barriers and facilitators for engagement and implementation of exercise in end-stage kidney disease: Future theory-based interventions using the Behavior Change Wheel. Semin Dial. 2019;32(4):308-19. DOI:10.1111/sdi.12787 | 3. Setting (not PC) |
| 37 | Clifford BK, Mizrahi D, Sandler CX, Barry BK, Simar D, Wakefield CE, et al. Barriers and facilitators of exercise experienced by cancer survivors: a mixed methods systematic review. Support Care Cancer. 2018;26(3):685-700. DOI:10.1007/s00520-017-3964-5 | 3. Setting (not PC) |
| 38 | Coffey L, Mooney O, Dunne S, Sharp L, Timmons A, Desmond D, et al. Perceived barriers and facilitators of participation in self-management interventions for cancer patients: A systematic review and meta-synthesis of qualitative studies. Supportive Care in Cancer. 2015;23(1):S321-S2. DOI:10.1007/s00520-015-2712-y | 1. Study type (not SR) |
| 39 | Cooper M, Avery L, Scott J, Ashley K, Jordan C, Errington L, et al. Effectiveness and active ingredients of social prescribing interventions targeting mental health: a systematic review. BMJ Open. 2022;12(7):e060214. DOI:10.1136/bmjopen-2021-060214 | 4. Outcome (not B+F) |
| 40 | Cox NS, Oliveira CC, Lahham A, Holland AE. Pulmonary rehabilitation referral and participation are commonly influenced by environment, knowledge, and beliefs about consequences: a systematic review using the Theoretical Domains Framework. Journal of Physiotherapy (Elsevier). 2017;63(2):84-93. DOI:10.1016/j.jphys.2017.02.002 | 3. Setting (not PC) |
| 41 | Crisford P, Winzenberg T, Aitken D, Schultz M, Cleland V. Factors associated with physical activity promotion in the allied health setting: A systematic review. Journal of Science and Medicine in Sport. 2015;19:e83. DOI:10.1016/j.jsams.2015.12.200 | 1. Study type (not SR) |
| 42 | Cunningham KB, Rogowsky RH, Carstairs SA, Sullivan F, Ozakinci G. Methods of connecting primary care patients with community-based physical activity opportunities: A realist scoping review. Health Soc Care Community. 2021;29(4):1169-99. DOI:10.1111/hsc.13186 | 5. Condition (not chronic) |
| 43 | Daly J, Sindone AP, Thompson DR, Hancock K, Chang E, Davidson P. Barriers to participation in and adherence to cardiac rehabilitation programs: a critical literature review. Prog Cardiovasc Nurs. 2002;17(1):8-17. DOI:10.1111/j.0889-7204.2002.00614.x | 1. Study type (not SR) |
| 44 | Darley A, Coughlan B, Furlong E. People with cancer and their family caregivers' personal experience of using supportive eHealth technology: A narrative review. European Journal of Oncology Nursing. 2021;54:N.PAG-N.PAG. DOI:10.1016/j.ejon.2021.102030 | 4. Outcome (not B+F) |
| 45 | Davenport S, Dickinson A, Minns Lowe C. Therapy-based exercise from the perspective of adult patients: a qualitative systematic review conducted using an ethnographic approach. Clin Rehabil. 2019;33(12):1963-77. DOI:10.1177/0269215519868797 | 3. Setting (not PC) |
| 46 | Davies F, Shepherd HL, Beatty L, Clark B, Butow P, Shaw J. Implementing web-based therapy in routine mental health care: Systematic review of health professionals' perspectives. Journal of Medical Internet Research. 2020;22(7). DOI:10.2196/17362 | 3. Setting (not PC) |
| 47 | Davies F, Wood F, Bullock A, Wallace C, Edwards A. Shifting mindsets: a realist synthesis of evidence from self‐management support training. Medical Education. 2018;52(3):274-87. DOI:10.1111/medu.13492 | 2. Intervention (not NDI) |
| 48 | de Oliveira NLZ, Peduzzi M, Agreli HLF, dos Santos Matsumoto K. Implementation of evidence-based nutritional management in primary health care settings: a systematic scoping review. Australian Journal of Primary Health. 2022;28(1):1-17. DOI:10.1071/PY20280 | 5. Condition (not chronic) |
| 49 | Deek H, Hamilton S, Brown N, Inglis SC, Digiacomo M, Newton PJ, et al. Family-centred approaches to healthcare interventions in chronic diseases in adults: a quantitative systematic review. J Adv Nurs. 2016;72(5):968-79. DOI:10.1111/jan.12885 | 4. Outcome (not B+F) |
| 50 | Dineen-Griffin S, Garcia-Cardenas V, Williams K, Benrimoj SI. Enhancing self-management support in primary health care: A systematic review of randomized controlled trials. Pharmacy Practice. 2018;16. DOI:10.18549/PharmPract.2018.s1.1338 | 1. Study type (not SR) |
| 51 | Dineen-Griffin S, Garcia-Cardenas V, Williams K, Benrimoj SI. Helping patients help themselves: A systematic review of self-management support strategies in primary health care practice. PLoS ONE Vol 14(8), 2019, ArtID e0220116. 2019;14(8). DOI:https://dx.doi.org/10.1371/journal.pone.0220116 | 4. Outcome (not B+F) |
| 52 | Dwarswaard J, Bakker EJ, van Staa A, Boeije HR. Self-management support from the perspective of patients with a chronic condition: a thematic synthesis of qualitative studies. Health Expect. 2016;19(2):194-208. DOI:10.1111/hex.12346 | 3. Setting (not PC) |
| 53 | Egerton T, Diamond LE, Buchbinder R, Bennell KL, Slade SC. A systematic review and evidence synthesis of qualitative studies to identify primary care clinicians' barriers and enablers to the management of osteoarthritis. Osteoarthritis Cartilage. 2017;25(5):625-38. DOI:10.1016/j.joca.2016.12.002 | 2. Intervention (not NDI) |
| 54 | Eisner D, Zoller M, Rosemann T, Huber CA, Badertscher N, Tandjung R. Screening and prevention in Swiss primary care: a systematic review. Int J Gen Med. 2011;4:853-70. DOI:10.2147/ijgm.s26562 | 2. Intervention (not NDI) |
| 55 | Evett D, Hutchinson K, Bierbaum M, Perikic N, Proctor C, Rapport F, et al. Peer support and social network groups among people living with epilepsy: A scoping review. Epilepsy Behav. 2021;124:108381. DOI:10.1016/j.yebeh.2021.108381 | 2. Intervention (not NDI) |
| 56 | Eyles JP, Ferreira ML, Rankin N, Salles A, March MK, Ferreira PH, et al. WHICH STRATEGIES ARE EFFECTIVE IN CHANGING HEALTH PROFESSIONAL BEHAVIOR ASSOCIATED WITH NON-PHARMACOLOGICAL, NON-SURGICAL MANAGEMENT OF OSTEOARTHRITIS AND SPINAL PAIN? A SYSTEMATIC REVIEW. Osteoarthritis and Cartilage. 2022;30:S399-S400. DOI:10.1016/j.joca.2022.02.537 | 1. Study type (not SR) |
| 57 | Featherstone C, Sharpe RA, Axford N, Asthana S, Husk K. Health and wellbeing outcomes and social prescribing pathways in community‐based support for autistic adults: A systematic mapping review of reviews. Health & Social Care in the Community. 2022;30(3):e621-e35. DOI:10.1111/hsc.13635 | 3. Setting (not PC) |
| 58 | Foss C, Knutsen I, Kennedy A, Todorova E, Wensing M, Lionis C, et al. Connectivity, contest and the ties of self-management support for type 2 diabetes: a meta-synthesis of qualitative literature. Health Soc Care Community. 2016;24(6):672-86. DOI:10.1111/hsc.12272 | 3. Setting (not PC) |
| 59 | Franklin M, Lewis S, Willis K, Bourke-Taylor H, Smith L. Patients' and healthcare professionals' perceptions of self-management support interactions: Systematic review and qualitative synthesis. Chronic Illn. 2018;14(2):79-103. DOI:10.1177/1742395317710082 | 3. Setting (not PC) |
| 60 | Frost R, Bhanu C, Walters K, Beattie A, Ben-Shlomo Y. Management of depression and referral of older people to psychological therapies: A systematic review of qualitative studies. British Journal of General Practice. 2019;69(680):E171-E81. DOI:10.3399/bjgp19X701297 | 3. Setting (not PC) |
| 61 | Fu Y, McNichol E, Marczewski K, Closs S. Patient-professional partnerships and chronic back pain self-management: A qualitative systematic review and synthesis. Health & Social Care in the Community. 2016;24(3):247-59. DOI:https://dx.doi.org/10.1111/hsc.12223 | 4. Outcome (not B+F) |
| 62 | Galdas P, Darwin Z, Fell J, Kidd L, Bower P, Blickem C, et al. Health Services and Delivery Research. A systematic review and metaethnography to identify how effective, cost-effective, accessible and acceptable self-management support interventions are for men with long-term conditions (SELF-MAN)2015. | 3. Setting (not PC) |
| 63 | Gaskins NJ, Bray EP, Hill JE, Doherty PJ, Harrison AS, Connell LA. Factors influencing implementation of aerobic exercise after stroke: a systematic review. Physiotherapy (United Kingdom). 2020;107:e163-e4. DOI:10.1016/j.physio.2020.03.239 | 3. Setting (not PC) |
| 64 | Gidlow C, Johnston LH, Crone D, James D. Attendance of exercise referral schemes in the UK: A systematic review. Health Education Journal. 2005;64(2):168-86. | 4. Outcome (not B+F) |
| 65 | Gil I, Santos-Costa P, Bobrowicz-Campos E, Santos E, Silva RC, Almeida MDL, et al. Group-based reminiscence interventions in older adults' with dementia assisted by technological innovations: A scoping review. European Geriatric Medicine. 2020;11(SUPPL 1):S237-S8. DOI:10.1007/s41999-020-00428-6 | 1. Study type (not SR) |
| 66 | González Moller J, Heaphy G, Urrutia Ortiz J. The Feasibility of Systemic Interventions for the Prevention and Treatment of Children and Adolescent Mental Health Difficulties in Latin American Countries: A Mixed Studies Systematic Review. Journal of Family Therapy. 2021;43(4):576-620. DOI:10.1111/1467-6427.12327 | 3. Setting (not PC) |
| 67 | Goodwin R, Hendrick P, Moffatt F, Logan P. First point of contact physiotherapy for patients with musculoskeletal conditions: how do professional issues influence implementation? A realist review. Physiotherapy (United Kingdom). 2019;105:e89-e90. DOI:10.1016/j.physio.2018.11.062 | 1. Study type (not SR) |
| 68 | Greenwell K, Sivyer K, Vedhara K, Yardley L, Game F, Chalder T, et al. Intervention planning for the REDUCE maintenance intervention: a digital intervention to reduce reulceration risk among patients with a history of diabetic foot ulcers. BMJ Open. 2018;8(5):e019865. DOI:10.1136/bmjopen-2017-019865 | 1. Study type (not SR) |
| 69 | Gross J, Vancampfort D, Stubbs B, Gorczynski P, Soundy A. A narrative synthesis investigating the use and value of social support to promote physical activity among individuals with schizophrenia. Disabil Rehabil. 2016;38(2):123-50. DOI:10.3109/09638288.2015.1024343 | 3. Setting (not PC) |
| 70 | Hall A, Richmond H, Copsey B, Hansen Z, Williamson E, Jones G, et al. Physiotherapist-delivered cognitive-behavioural interventions are effective for low back pain, but can they be replicated in clinical practice? A systematic review. Disabil Rehabil. 2018;40(1):1-9. DOI:10.1080/09638288.2016.1236155 | 4. Outcome (not B+F) |
| 71 | Hall LH, Thorneloe R, Rodriguez-Lopez R, Grice A, Thorat MA, Bradbury K, et al. Delivering brief physical activity interventions in primary care: a systematic review. Br J Gen Pract. 2022;72(716):e209-e16. DOI:10.3399/bjgp.2021.0312 | 5. Condition (not chronic) |
| 72 | Heath JM, Stuart MR. Prescribing exercise for frail elders. J Am Board Fam Pract. 2002;15(3):218-28. | 1. Study type (not SR) |
| 73 | Hébert ET, Caughy MO, Shuval K. Primary care providers' perceptions of physical activity counselling in a clinical setting: a systematic review. Br J Sports Med. 2012;46(9):625-31. DOI:10.1136/bjsports-2011-090734 | 5. Condition (not chronic) |
| 74 | Herrera S, Salazar A, Nazar G. Barriers and Supports in eHealth Implementation among People with Chronic Cardiovascular Ailments: Integrative Review. Int J Environ Res Public Health. 2022;19(14). DOI:10.3390/ijerph19148296 | 2. Intervention (not NDI) |
| 75 | Holm AL, Severinsson E. Chronic care model for the management of depression: Synthesis of barriers to, and facilitators of, success. International Journal of Mental Health Nursing. 2012;21(6):513-23. DOI:10.1111/j.1447-0349.2012.00827.x | 2. Intervention (not NDI) |
| 76 | Huijg J, Gebhardt W, Verheijden M, Zouwe N, Vries J, Middelkoop B, et al. Factors Influencing Primary Health Care Professionals' Physical Activity Promotion Behaviors: A Systematic Review. International Journal of Behavioral Medicine. 2015;22(1):32-50. DOI:10.1007/s12529-014-9398-2 | 5. Condition (not chronic) |
| 77 | Hurley J, O'Keeffe M, Synnott A, Bunzli S, Dankaerts W, O'Sullivan P, et al. To investigate patient beliefs regarding low back pain (LBP) following conservative physical rehabilitation: A systematic review. Manual Therapy. 2016;25:e139-e40. DOI:10.1016/j.math.2016.05.265 | 1. Study type (not SR) |
| 78 | Hurley M, Dickson K, Hauari H, Walsh NE, Grant R, Cumming J, et al. People's views, beliefs and experiences of exercise for chronic hip and knee pain: Cochrane review with qualitative synthesis. Arthritis and Rheumatology. 2014;66:S1017. DOI:10.1002/art.38914 | 1. Study type (not SR) |
| 79 | IJsbrandy C, Ottevanger PB, Tsekou Diogeni M, Gerritsen WR, van Harten WH, Hermens R. Review: Effectiveness of implementation strategies to increase physical activity uptake during and after cancer treatment. Crit Rev Oncol Hematol. 2018;122:157-63. DOI:10.1016/j.critrevonc.2017.09.005 | 3. Setting (not PC) |
| 80 | Ince P, Haddock G, Tai S. A systematic review of the implementation of recommended psychological interventions for schizophrenia: Rates, barriers, and improvement strategies. Psychology & Psychotherapy: Theory, Research & Practice. 2016;89(3):324-50. DOI:10.1111/papt.12084 | 3. Setting (not PC) |
| 81 | Jack K, McLean SM, Moffett JK, Gardiner E. Barriers to treatment adherence in physiotherapy outpatient clinics: a systematic review. Man Ther. 2010;15(3):220-8. DOI:10.1016/j.math.2009.12.004 | 3. Setting (not PC) |
| 82 | Jain SR, Sui Y, Ng CH, Chen ZX, Goh LH, Shorey S. Patients' and healthcare professionals' perspectives towards technology-assisted diabetes self-management education. A qualitative systematic review. PLoS One. 2020;15(8):e0237647. DOI:10.1371/journal.pone.0237647 | 3. Setting (not PC) |
| 83 | Jakubowski B, Tucker K, Hinton L, Jaspreet K, McManus R. Is self-management a burden? A systematic review of the experiences of women self-managing chronic conditions during pregnancy. BJOG: An International Journal of Obstetrics and Gynaecology. 2020;127(8):e65-e6. DOI:10.1111/1471-0528.16274 | 1. Study type (not SR) |
| 84 | Jakubowski BE, Hinton L, Khaira J, Roberts N, McManus RJ, Tucker KL. Is self-management a burden? What are the experiences of women self-managing chronic conditions during pregnancy? A systematic review. BMJ Open. 2022;12(3):e051962. DOI:10.1136/bmjopen-2021-051962 | 3. Setting (not PC) |
| 85 | Jimison H, Gorman P, Woods S, Nygren P, Walker M, Norris S, et al. Barriers and drivers of health information technology use for the elderly, chronically ill, and underserved. Evid Rep Technol Assess (Full Rep). 2008(175):1-1422. | 5. Condition (not chronic) |
| 86 | Jordan RE, Majothi S, Heneghan NR, Blissett DB, Riley RD, Sitch AJ, et al. Supported self-management for patients with moderate to severe chronic obstructive pulmonary disease (COPD): an evidence synthesis and economic analysis. Health Technol Assess. 2015;19(36):1-516. DOI:10.3310/hta19360 | 3. Setting (not PC) |
| 87 | Joseph R, Hart NH, Bradford N, Agbejule OA, Koczwara B, Chan A, et al. Diet and exercise advice and referrals for cancer survivors: an integrative review of medical and nursing perspectives. Support Care Cancer. 2022. DOI:10.1007/s00520-022-07152-w | 3. Setting (not PC) |
| 88 | Kantilal K, Hardeman W, Whiteside H, Karapanagiotou E, Small M, Bhattacharya D. Facilitating healthcare practitioners to deliver self-management support in adult cancer survivors: A realist review. Res Social Adm Pharm. 2022. DOI:10.1016/j.sapharm.2022.05.011 | 3. Setting (not PC) |
| 89 | Kantilal K. Facilitating healthcare practitioners to deliver self-management support in adult cancer survivors: a realist review. Journal of Oncology Pharmacy Practice. 2022;28(2 SUPPL):13-4. DOI:10.1177/10781552221078082 | 1. Study type (not SR) |
| 90 | Khan N, Bower P, Rogers A. Guided self-help in primary care mental health: meta-synthesis of qualitative studies of patient experience. Br J Psychiatry. 2007;191:206-11. DOI:10.1192/bjp.bp.106.032011 | 4. Outcome (not B+F) |
| 91 | Kirby SE, Miles C, Arden-Close E, Yardley L, Bruton A, Hankins M, et al. Barriers and facilitators to effective self-management of asthma-a systematic review and thematic synthesis. Thorax. 2014;69:A96. DOI:10.1136/thoraxjnl-2014-206260.192 | 1. Study type (not SR) |
| 92 | Kousoulis AA, Patelarou E, Shea S, Foss C, Ruud Knutsen IA, Todorova E, et al. Diabetes self-management arrangements in Europe: a realist review to facilitate a project implemented in six countries. BMC Health Serv Res. 2014;14:453. DOI:10.1186/1472-6963-14-453 | 3. Setting (not PC) |
| 93 | Leite Soares V, Queiroz de Carvalho Gomes M, Medeiros de Sousa JM, Soares Carvalho P, Barros Palitot E, Guimarães Oliveira Soares MJ. EXPLORANDO EVIDÊNCIAS CIENTÍFICAS SOBRE AÇÕES DE AUTOCUIDADO EM PACIENTES COM PSORÍASE: REVISÃO INTEGRATIVA. Enfermagem Atual in Derme. 2021;95(36):1-19. DOI:10.31011/reaid-2021-v.95-n.36-art.1201 | 3. Setting (not PC) |
| 94 | Li C-C, Shun S-C. Understanding self care coping styles in patients with chronic heart failure: A systematic review. European Journal of Cardiovascular Nursing. 2016;15(1):12-9. DOI:10.1177/1474515115572046 | 2. Intervention (not NDI) |
| 95 | Lightfoot C, Wilkinson T, Nixon D, Song Y, Smith A. Barriers and benefits to engagement in exercise in peritoneal dialysis patients. Nephrology Dialysis Transplantation. 2019;34:a527. DOI:10.1093/ndt/gfz103.SP504 | 1. Study type (not SR) |
| 96 | Little M, Rosa E, Heasley C, Asif A, Dodd W, Richter A. Promoting Healthy Food Access and Nutrition in Primary Care: A Systematic Scoping Review of Food Prescription Programs. Am J Health Promot. 2022;36(3):518-36. DOI:10.1177/08901171211056584 | 5. Condition (not chronic) |
| 97 | Liu Y, Gellatly J. Barriers and facilitators of engagement in psychological therapies among older adults with depression: A systematic review and thematic synthesis. Journal of Psychiatric & Mental Health Nursing (John Wiley & Sons, Inc). 2021;28(4):509-20. DOI:10.1111/jpm.12697 | 3. Setting (not PC) |
| 98 | Macdonald EM, Perrin BM, Kingsley MI. Enablers and barriers to using two-way information technology in the management of adults with diabetes: A descriptive systematic review. J Telemed Telecare. 2018;24(5):319-40. DOI:10.1177/1357633x17699990 | 2. Intervention (not NDI) |
| 99 | Majeed R, Jackson C, Cheater FM, Knapp P. A systematic review of research into Black and minority ethnic patients' views on selfmanagement of Type 2 diabetes. Diabetic Medicine. 2013;30:19. DOI:10.1111/dme.12090_11 | 1. Study type (not SR) |
| 100 | Majeed-Ariss R, Jackson C, Knapp P, Cheater FM. A systematic review of research into black and ethnic minority patients' views on self-management of type 2 diabetes. Health Expect. 2015;18(5):625-42. DOI:10.1111/hex.12080 | 3. Setting (not PC) |
| 101 | Mansfield E, Noble N, Sanson-Fisher R, Mazza D, Bryant J. Primary Care Physicians' Perceived Barriers to Optimal Dementia Care: A Systematic Review. Gerontologist. 2019;59(6):e697-e708. DOI:10.1093/geront/gny067 | 2. Intervention (not NDI) |
| 102 | Martin Ginis KA, Ma JK, Latimer-Cheung AE, Rimmer JH. A systematic review of review articles addressing factors related to physical activity participation among children and adults with physical disabilities. Health Psychol Rev. 2016;10(4):478-94. DOI:10.1080/17437199.2016.1198240 | 3. Setting (not PC) |
| 103 | McCurdy AP, Lamboglia CG, Lindeman C, Mangan A, Wohlers B, Sivak A, et al. The physical activity sector within the treatment of mental illness: A scoping review of the perceptions of healthcare professionals. Mental Health and Physical Activity. 2020;19. DOI:10.1016/j.mhpa.2020.100349 | 3. Setting (not PC) |
| 104 | Meredith SJ, Cox N, Ibrahim K, Higson J, McNiff J, Rutherford M, et al. FACTORS THAT INFLUENCE OLDER ADULTS' PARTICIPATION IN PHYSICAL ACTIVITY: A SYSTEMATIC REVIEW OF QUALITATIVE STUDIES. Age and Ageing. 2022;51:ii5. DOI:10.1093/ageing/afac125 | 1. Study type (not SR) |
| 105 | Meshe OF, Claydon LS, Bungay H, Andrew S. The relationship between physical activity and health status in patients with chronic obstructive pulmonary disease following pulmonary rehabilitation. Disabil Rehabil. 2017;39(8):746-56. DOI:10.3109/09638288.2016.1161842 | 3. Setting (not PC) |
| 106 | Meyer C, Hill S, Dow B, Synnot A, Hill K. Translating Falls Prevention Knowledge to Community-Dwelling Older PLWD: A Mixed-Method Systematic Review. Gerontologist. 2015;55(4):560-74. DOI:10.1093/geront/gnt127 | 2. Intervention (not NDI) |
| 107 | Mileski M, Kruse CS, Catalani J, Haderer T. Adopting Telemedicine for the Self-Management of Hypertension: Systematic Review. JMIR Med Inform. 2017;5(4):e41. DOI:10.2196/medinform.6603 | 2. Intervention (not NDI) |
| 108 | Mlenzana NB, Frantz JM, Rhoda AJ, Eide AH. Barriers to and facilitators of rehabilitation services for people with physical disabilities: A systematic review. Afr J Disabil. 2013;2(1):22. DOI:10.4102/ajod.v2i1.22 | 2. Intervention (not NDI) |
| 109 | Moncion K, Biasin L, Jagroop D, Bayley M, Danells C, Mansfield A, et al. Barriers and Facilitators to Aerobic Exercise Implementation in Stroke Rehabilitation: A Scoping Review. J Neurol Phys Ther. 2020;44(3):179-87. DOI:10.1097/npt.0000000000000318 | 3. Setting (not PC) |
| 110 | Moore DA, Nunns M, Shaw L, Rogers M, Walker E, Ford T, et al. Interventions to improve the mental health of children and young people with long-term physical conditions: linked evidence syntheses. Health Technol Assess. 2019;23(22):1-164. DOI:10.3310/hta23220 | 3. Setting (not PC) |
| 111 | Moore SA, Flynn D, Price CI, Avery L. Using intervention mapping to develop and facilitate implementation of a multifaceted behavioural intervention targeting physical activity and sedentary behaviour in stroke survivors: Physical Activity Routines After Stroke (PARAS): Intervention development study. Health Psychology and Behavioral Medicine. 2022;10(1):439-66. DOI:https://dx.doi.org/10.1080/21642850.2022.2066534 | 3. Setting (not PC) |
| 112 | Morgan F, Battersby A, Weightman AL, Searchfield L, Turley R, Morgan H, et al. Adherence to exercise referral schemes by participants - what do providers and commissioners need to know? A systematic review of barriers and facilitators. BMC Public Health. 2016;16:227. DOI:10.1186/s12889-016-2882-7 | 5. Condition (not chronic) |
| 113 | Morris JH, Irvine LA, Dombrowski SU, McCormack B, Van Wijck F, Lawrence M. We Walk: a person-centred, dyadic behaviour change intervention to promote physical activity through outdoor walking after stroke-an intervention development study. BMJ Open. 2022;12(6):e058563. DOI:10.1136/bmjopen-2021-058563 | 1. Study type (not SR) |
| 114 | Morris ME, Slade SC, Bruce C, McGinley JL, Bloem BR. Enablers to Exercise Participation in Progressive Supranuclear Palsy: Health Professional Perspectives. Front Neurol. 2020;11:635341. DOI:10.3389/fneur.2020.635341 | 1. Study type (not SR) |
| 115 | Murray J, Craigs CL, Hill KM, Honey S, House A. A systematic review of patient reported factors associated with uptake and completion of cardiovascular lifestyle behaviour change. BMC Cardiovasc Disord. 2012;12:120. DOI:10.1186/1471-2261-12-120 | 4. Outcome (not B+F) |
| 116 | Murray J, Honey S, Hill K, Craigs C, House A. Individual influences on lifestyle change to reduce vascular risk: a qualitative literature review. Br J Gen Pract. 2012;62(599):e403-10. DOI:10.3399/bjgp12X649089 | 3. Setting (not PC) |
| 117 | Neale EP, Middleton J, Lambert K. Barriers and enablers to detection and management of chronic kidney disease in primary healthcare: a systematic review. BMC Nephrol. 2020;21(1):83. DOI:10.1186/s12882-020-01731-x | 2. Intervention (not NDI) |
| 118 | Neelakantan L, Hetrick S, Michelson D. Users' experiences of trauma-focused cognitive behavioural therapy for children and adolescents: a systematic review and metasynthesis of qualitative research. Eur Child Adolesc Psychiatry. 2019;28(7):877-97. DOI:10.1007/s00787-018-1150-z | 5. Condition (not chronic) |
| 119 | Neri M, Spanevello A. Review of self-management in asthmatics. European Respiratory Review. 1993;3(14):408-9. | 1. Study type (not SR) |
| 120 | Neven ACH, Lake AJ, Williams A, O'Reilly SL, Hendrieckx C, Morrison M, et al. Barriers to and enablers of postpartum health behaviours among women from diverse cultural backgrounds with prior gestational diabetes: a systematic review and qualitative synthesis applying the Theoretical Domains Framework. Diabet Med. 2022:e14945. DOI:10.1111/dme.14945 | 5. Condition (not chronic) |
| 121 | Niño de Guzmán Quispe E, Martínez García L, Orrego Villagrán C, Heijmans M, Sunol R, Fraile-Navarro D, et al. The Perspectives of Patients with Chronic Diseases and Their Caregivers on Self-Management Interventions: A Scoping Review of Reviews. Patient. 2021;14(6):719-40. DOI:10.1007/s40271-021-00514-2 | 6. Duplicate |
| 122 | Nino de Guzman Quispe E, Martinez Garcia L, Orrego Villagran C, Heijmans M, Sunol R, Fraile-Navarro D, et al. The perspectives of patients with chronic diseases and their caregivers on self-management interventions: A scoping review of reviews. The Patient: Patient-Centered Outcomes Research. 2021:No Pagination Specified. DOI:https://dx.doi.org/10.1007/s40271-021-00514-2 | 1. Study type (not SR) |
| 123 | Nissen N, Holm PM, Bricca A, Dideriksen M, Tang LH, Skou ST. Clinicians' beliefs and attitudes to physical activity and exercise therapy as treatment for knee and/or hip osteoarthritis: a scoping review. Osteoarthritis Cartilage. 2022;30(2):260-9. DOI:10.1016/j.joca.2021.11.008 | 4. Outcome (not B+F) |
| 124 | Nixon AL, Leonardi-Bee J, Haiquan W, Chattopadhyay K. Barriers and facilitators to type 2 diabetes management in the Caribbean region: a qualitative systematic review. JBI Evidence Synthesis. 2021;19(5):911-65. DOI:10.11124/JBISRIR-D-19-00424 | 2. Intervention (not NDI) |
| 125 | O'Connell S, McCarthy VJ, Savage E. Self-management support preferences of people with asthma or chronic obstructive pulmonary disease: A systematic review and meta-synthesis of qualitative studies. Chronic Illn. 2021;17(3):283-305. DOI:10.1177/1742395319869443 | 3. Setting (not PC) |
| 126 | Oliver EM, Adams AE. Influences on dietary and lifestyle changes among people with type 2 diabetes. Diabetic Medicine. 2010;27(2):116. DOI:10.1111/j.1464-5491.2009.02936.x | 1. Study type (not SR) |
| 127 | O'Shea A, Drennan J, Littlewood C, Slater H, Sim J, McVeigh J. Barriers and facilitators related to self management of shoulder pain: A systematic review and qualitative synthesis. Annals of the Rheumatic Diseases. 2021;80(SUPPL 1):293. DOI:10.1136/annrheumdis-2021-eular.1279 | 1. Study type (not SR) |
| 128 | Palacholla RS, Fischer N, Coleman A, Agboola S, Kirley K, Felsted J, et al. Provider- and Patient-Related Barriers to and Facilitators of Digital Health Technology Adoption for Hypertension Management: Scoping Review. JMIR Cardio. 2019;3(1):e11951. DOI:10.2196/11951 | 3. Setting (not PC) |
| 129 | Perez A. Self-management of hypertension in Hispanic adults. Clin Nurs Res. 2011;20(4):347-65. DOI:10.1177/1054773811411582 | 3. Setting (not PC) |
| 130 | Petter M, Blanchard C, Kemp KA, Mazoff AS, Ferrier SN. Correlates of exercise among coronary heart disease patients: review, implications and future directions. Eur J Cardiovasc Prev Rehabil. 2009;16(5):515-26. DOI:10.1097/HJR.0b013e3283299585 | 3. Setting (not PC) |
| 131 | Phiri T, Mowat R, Cook C. What nursing interventions and healthcare practices facilitate type 1 diabetes self-management in young adults? An integrative review. Nursing Praxis in Aotearoa New Zealand. 2022;38(2):32-43. DOI:10.36951/27034542.2022.12 | 3. Setting (not PC) |
| 132 | Pugh P, Hemingway P, Christian M, Higginbottom G. Children's, parents', and other stakeholders' perspectives on the factors influencing the initiation of early dietary change in the management of childhood chronic disease: A mixed studies systematic review using a narrative synthesis. Patient Education and Counseling. 2020:No Pagination Specified. DOI:https://dx.doi.org/10.1016/j.pec.2020.09.021 | 3. Setting (not PC) |
| 133 | Pugh P, Hemingway P, Christian M, Higginbottom G. Children's, parents', and other stakeholders' perspectives on the factors influencing the initiation of early dietary change in the management of childhood chronic disease: a mixed studies systematic review using a narrative synthesis. Patient Educ Couns. 2021;104(4):844-57. DOI:10.1016/j.pec.2020.09.021 | 6. Duplicate |
| 134 | Rees J, Cooper C, Burton A, Tuijt R, Walters K. Self-care of long term conditions in dementia: Systematic review of factors affecting self or proxy management. International Psychogeriatrics. 2019;31:72. DOI:10.1017/S1041610219001339 | 1. Study type (not SR) |
| 135 | Rees S, Williams A. Promoting and supporting self-management for adults living in the community with physical chronic illness: A systematic review of the effectiveness and meaningfulness of the patient-practitioner encounter. JBI Libr Syst Rev. 2009;7(13):492-582. DOI:10.11124/01938924-200907130-00001 | 4. Outcome (not B+F) |
| 136 | Riggs DM, Killingback C. What factors influence physical activity participation in people with rheumatoid arthritis? Physical Therapy Reviews. 2019;24(6):298-307. DOI:10.1080/10833196.2019.1659562 | 3. Setting (not PC) |
| 137 | Roberts SH, Bailey JE. Incentives and barriers to lifestyle interventions for people with severe mental illness: A narrative synthesis of quantitative, qualitative and mixed methods studies. Journal of Advanced Nursing. 2011;67(4):690-708. DOI:https://dx.doi.org/10.1111/j.1365-2648.2010.05546.x | 1. Study type (not SR) |
| 138 | Robinson H, Hill E, Peel J, Direito A, Jones AW. Patient and public involvement and application of the behaviour change wheel to promote physical activity following pulmonary rehabilitation in COPD: An intervention development study. European Respiratory Journal. 2019;54. DOI:10.1183/13993003.congress-2019.PA653 | 1. Study type (not SR) |
| 139 | Robinson H, Williams V, Curtis F, Bridle C, Jones AW. Facilitators and barriers to physical activity following pulmonary rehabilitation in COPD: a systematic review of qualitative studies. NPJ Prim Care Respir Med. 2018;28(1):19. DOI:10.1038/s41533-018-0085-7 | 3. Setting (not PC) |
| 140 | Rosenkranz RR, Kolt GS, Brown J, Berentson-Shaw J. A review of enablers and barriers to physical activity participation among older people of New Zealand and international populations. International SportMed Journal. 2013;14(4):294-312. | 5. Condition (not chronic) |
| 141 | Russell S, Ogunbayo OJ, Newham JJ, Heslop-Marshall K, Netts P, Hanratty B, et al. Qualitative systematic review of barriers and facilitators to self-management of chronic obstructive pulmonary disease: views of patients and healthcare professionals. NPJ Prim Care Respir Med. 2018;28(1):2. DOI:10.1038/s41533-017-0069-z | 3. Setting (not PC) |
| 142 | Sadler EA, Wolfe CDA, McKevitt C. Patients' and health professionals' views of self-management: Systematic review and narrative synthesis. Cerebrovascular Diseases. 2013;35:772. DOI:10.1159/000353129 | 1. Study type (not SR) |
| 143 | Slade PP, Molyneux DR, Watt DA. A systematic review of clinical effectiveness of psychological interventions to reduce post traumatic stress symptoms following childbirth and a meta-synthesis of facilitators and barriers to uptake of psychological care. J Affect Disord. 2021;281:678-94. DOI:10.1016/j.jad.2020.11.092 | 3. Setting (not PC) |
| 144 | Sohanpal R, Steed EA, Taylor SJC. Understanding reasons for patient attendance and non-attendance in pulmonary rehabilitation and COPD self-management programmes. A qualitative synthesis and application of theory. Thorax. 2012;67:A54. DOI:10.1136/thoraxjnl-2012-202678.116 | 1. Study type (not SR) |
| 145 | Somerville M, Ball L, Sierra-Silvestre E, Williams LT. Understanding the knowledge, attitudes and practices of providing and receiving nutrition care for prediabetes: an integrative review. Aust J Prim Health. 2019;25(4):289-302. DOI:10.1071/py19082 | 4. Outcome (not B+F) |
| 146 | Sonderlund AL, Thilsing T, Korevaar J, Hollander M, Lionis C, Schellevis F, et al. An evidence-based toolbox for the design and implementation of selective-prevention primary-care initiatives targeting cardio-metabolic disease. Preventive Medicine Reports. 2019;16. DOI:10.1016/j.pmedr.2019.100979 | 1. Study type (not SR) |
| 147 | Soundy A, Freeman P, Stubbs B, Probst M, Coffee P, Vancampfort D. The transcending benefits of physical activity for individuals with schizophrenia: a systematic review and meta-ethnography. Psychiatry Res. 2014;220(1-2):11-9. DOI:10.1016/j.psychres.2014.07.083 | 3. Setting (not PC) |
| 148 | Spaling MA, Currie K, Strachan PH, Harkness K, Clark AM. Improving support for heart failure patients: a systematic review to understand patients' perspectives on self-care. J Adv Nurs. 2015;71(11):2478-89. DOI:10.1111/jan.12712 | 3. Setting (not PC) |
| 149 | Spink A, Wagner I, Orrock P. Common reported barriers and facilitators for self-management in adults with chronic musculoskeletal pain: A systematic review of qualitative studies. Musculoskelet Sci Pract. 2021;56:102433. DOI:10.1016/j.msksp.2021.102433 | 3. Setting (not PC) |
| 150 | Stenberg U, Haaland-Øverby M, Fredriksen K, Westermann KF, Kvisvik T. A scoping review of the literature on benefits and challenges of participating in patient education programs aimed at promoting self-management for people living with chronic illness. Patient Educ Couns. 2016;99(11):1759-71. DOI:10.1016/j.pec.2016.07.027 | 3. Setting (not PC) |
| 151 | Subedi N, Rawstorn JC, Gao L, Koorts H, Maddison R. Implementation of Telerehabilitation Interventions for the Self-Management of Cardiovascular Disease: Systematic Review. JMIR Mhealth Uhealth. 2020;8(11):e17957. DOI:10.2196/17957 | 3. Setting (not PC) |
| 152 | Svendsen MJ, Wood KW, Kyle J, Cooper K, Rasmussen CDN, Sandal LF, et al. Barriers and facilitators to patient uptake and utilisation of digital interventions for the self-management of low back pain: a systematic review of qualitative studies. BMJ Open. 2020;10(12):e038800. DOI:10.1136/bmjopen-2020-038800 | 3. Setting (not PC) |
| 153 | Swärdh E, Opava C, Brodin N. Physical activity in patients with rheumatoid arthritis - an agile lifelong behaviour: a qualitative meta-synthesis. RMD Open. 2021;7(2). DOI:10.1136/rmdopen-2021-001635 | 3. Setting (not PC) |
| 154 | Swift E, O'Brien MR, Peters S, Kelly C. Healthcare professionals' perceptions of pulmonary rehabilitation as a management strategy for patients with chronic obstructive pulmonary disease: a critical interpretive synthesis. Disabil Rehabil. 2022;44(4):520-35. DOI:10.1080/09638288.2020.1769745 | 3. Setting (not PC) |
| 155 | Tan CC, Cheng KK, Wang W. Self-care management programme for older adults with diabetes: An integrative literature review. Int J Nurs Pract. 2015;21 Suppl 2:115-24. DOI:10.1111/ijn.12388 | 4. Outcome (not B+F) |
| 156 | Tao L, Carboni-Jiménez A, Turner K, Østbø N, Aguila K, Boruff J, et al. Perceived Barriers and Facilitators of Using Synchronous Telerehabilitation of Physical and Occupational Therapy in Musculoskeletal Disorders: A Scoping Review. 2022. DOI:10.1101/2022.07.21.22277858 | 3. Setting (not PC) |
| 157 | Thorpe O, Johnston K, Kumar S. Barriers and enablers to physical activity participation in patients with COPD: a systematic review. J Cardiopulm Rehabil Prev. 2012;32(6):359-69. DOI:10.1097/HCR.0b013e318262d7df | 3. Setting (not PC) |
| 158 | Tian J, Zhou F, Zhang XG, Wang HY, Peng SH, Li X, et al. Experience of physical activity in patients with COPD: A systematic review and qualitative meta-synthesis. Geriatr Nurs. 2022;47:211-9. DOI:10.1016/j.gerinurse.2022.07.013 | 3. Setting (not PC) |
| 159 | Tierney S, Deaton C, Mamas M, Rutter M, Gibson M, Neyses L. Understanding barriers and enablers of physical activity among patients with heart failure: A systematic review of qualitative studies. European Journal of Cardiovascular Nursing. 2011;10:S28. DOI:10.1016/S1474-5151(11)60102-3 | 1. Study type (not SR) |
| 160 | Toback M, Clark N. Strategies to improve self-management in heart failure patients. Contemp Nurse. 2017;53(1):105-20. DOI:10.1080/10376178.2017.1290537 | 3. Setting (not PC) |
| 161 | Tuudah E, Foye U, Donetto S, Simpson A. Non-Pharmacological Integrated Interventions for Adults Targeting Type 2 Diabetes and Mental Health Comorbidity: A Mixed-Methods Systematic Review. Int J Integr Care. 2022;22(2):27. DOI:10.5334/ijic.5960 | 3. Setting (not PC) |
| 162 | van Dongen SI, de Nooijer K, Cramm JM, Francke AL, Oldenmenger WH, Korfage IJ, et al. Self-management of patients with advanced cancer: A systematic review of experiences and attitudes. Palliat Med. 2020;34(2):160-78. DOI:10.1177/0269216319883976 | 3. Setting (not PC) |
| 163 | Vancampfort D, Mugisha J, Richards J, De Hert M, Probst M, Stubbs B. Physical activity correlates in people living with HIV/AIDS: a systematic review of 45 studies. Disabil Rehabil. 2018;40(14):1618-29. DOI:10.1080/09638288.2017.1306587 | 4. Outcome (not B+F) |
| 164 | Veldhuijzen van Zanten J, Rouse P, Hale E, Ntoumanis N, Metsios G, Duda J, et al. Perceived Barriers, Facilitators and Benefits for Regular Physical Activity and Exercise in Patients with Rheumatoid Arthritis: A Review of the Literature. Sports Medicine. 2015;45(10):1401-12. DOI:10.1007/s40279-015-0363-2 | 3. Setting (not PC) |
| 165 | Vseteckova J, Dadova K, Gracia R, Ryan G, Borgstrom E, Abington J, et al. Barriers and facilitators to adherence to walking group exercise in older people living with dementia in the community: a systematic review. Eur Rev Aging Phys Act. 2020;17:15. DOI:10.1186/s11556-020-00246-6 | 4. Outcome (not B+F) |
| 166 | Williams TL, Smith B, Papathomas A. The barriers, benefits and facilitators of leisure time physical activity among people with spinal cord injury: A meta-synthesis of qualitative findings. Health Psychology Review. 2014;8(4):404-25. DOI:https://dx.doi.org/10.1080/17437199.2014.898406 | 2. Intervention (not NDI) |
| 167 | Wolker Manta S, Fabrício Sandreschi P, Christofoletti Dos Santos M, Maria Konrad L, Miranda Tassitano R, Rosane Bertoldo Benedetti T. Barriers and facilitators on the implementation of physical activity in Primary Health Care: A systematic review. Prev Med Rep. 2022;28:101875. DOI:10.1016/j.pmedr.2022.101875 | 5. Condition (not chronic) |
| 168 | Yannitsos D, Murphy RA, Pollock P, Di Sebastiano KM. Facilitators and barriers to participation in lifestyle modification for men with prostate cancer: A scoping review. Eur J Cancer Care (Engl). 2020;29(1):e13193. DOI:10.1111/ecc.13193 | 3. Setting (not PC) |
| ***Excluded articles from additional sources (included abstracts in FT screening)*** | | |
| 1 | Alaslawi H, Berrou I, Al Hamid A, Alhuwail D, Aslanpour Z. Diabetes Self-management Apps: Systematic Review of Adoption Determinants and Future Research Agenda. JMIR Diabetes. 2022;7(3):e28153. DOI:10.2196/28153 | 2. Intervention (not NDI) |
| 2 | Coffey L, Mooney O, Dunne S, Sharp L, Timmons A, Desmond D, et al. Cancer survivors' perspectives on adjustment-focused self-management interventions: a qualitative meta-synthesis. J Cancer Surviv. 2016;10(6):1012-34. DOI:10.1007/s11764-016-0546-3 | 5. Condition (not chronic) |
| 3 | Crisford P, Winzenberg T, Venn A, Schultz M, Aitken D, Cleland V. Factors associated with physical activity promotion by allied and other non-medical health professionals: A systematic review. Patient Educ Couns. 2018;101(10):1775-85. DOI:10.1016/j.pec.2018.05.011 | 3. Setting (not PC) |
| 4 | Crowley J, Ball L, Hiddink GJ. Nutrition care by primary-care physicians: advancing our understanding using the COM-B framework. Public Health Nutrition. 2020;23(1):41-52. DOI:10.1017/S1368980019003148 | 3. Setting (not PC) |
| 5 | Lightfoot CJ, Wilkinson TJ, Song Y, Burton JO, Smith AC. Perceptions of exercise benefits and barriers: the influence on physical activity behaviour in individuals undergoing haemodialysis and peritoneal dialysis. Journal of Nephrology. 2021;34(6):1961-71. DOI:10.1007/s40620-021-01024-y | 1. Study type (not SR) |
| 6 | Moore SA, Hrisos N, Flynn D, Errington L, Price C, Avery L. How should long-term free-living physical activity be targeted after stroke? A systematic review and narrative synthesis. Int J Behav Nutr Phys Act. 2018;15(1):100. DOI:10.1186/s12966-018-0730-0 | 3. Setting (not PC) |
| 7 | Morris J, Oliver T, Kroll T, Macgillivray S. The importance of psychological and social factors in influencing the uptake and maintenance of physical activity after stroke: a structured review of the empirical literature. Stroke Res Treat. 2012;2012:195249. DOI:10.1155/2012/195249 | 3. Setting (not PC) |
| 8 | O'Shea A, Drennan J, Littlewood C, Slater H, Sim J, McVeigh JG. Barriers and facilitators related to self-management of shoulder pain: a systematic review and qualitative synthesis. Clin Rehabil. 2022;36(11):1539-62. DOI:10.1177/02692155221108553 | 3. Setting (not PC) |
| 9 | Rees J, Tuijt R, Burton A, Walters K, Cooper C. Supporting self-care of long-term conditions in people with dementia: A systematic review. Int J Nurs Stud. 2021;116:103432. DOI:10.1016/j.ijnurstu.2019.103432 | 3. Setting (not PC) |
| 10 | Sohanpal R, Steed L, Mars T, Taylor SJ. Understanding patient participation behaviour in studies of COPD support programmes such as pulmonary rehabilitation and self-management: a qualitative synthesis with application of theory. NPJ Prim Care Respir Med. 2015;25:15054. DOI:10.1038/npjpcrm.2015.54 | 3. Setting (not PC) |
