# Supplementary material for: Common barriers and enablers to the use of non-drug interventions for managing common chronic conditions in primary care: an overview of reviews
Source: BMC Prim Care. 2024 Apr 6;25:108. doi: 10.1186/s12875-024-02321-8 (PMC10998330; doi:10.1186/s12875-024-02321-8)
Supplement: Supplementary file 5 — Supplementary Material 5. [file 12875_2024_2321_MOESM5_ESM.docx]

| **Additional file 5: Characteristics of Included Studies** | | | | | |  |  |  |  |  |
| --- | --- | --- | --- | --- | --- | --- | --- | --- | --- | --- |
| **NDI Type** | **First author, Year** | **Countries of studies** | **No. studies** | **No. Participants** | **Health condition of Participants** | **Reported NDI** | **Methods of studies** | **Analysis methods of studies** | **Review Analysis method** | **ROB tool** |
| ***Interventions to prevent chronic health conditions*** | | | | | | | | | | |
| ***Physical*** | Albert, 2020 | UK, Spain, Sweden, Denmark, Australia, Netherlands, New Zealand, USA | 27 | 15743 patients | Various chronic conditions (sedentary/inactive, cardiovascular,overweight/obesity, musculoskeletal, diabetes, psychological, smoker, cancer, stroke) | referred for individual or groups physical activity interventions (e.g., group supervised activities, counseling and advice, individualized supervised or unsupervised activity, referral to other HCPs) | Quant (RCTs, longitudinal, case, cohort, cross sectional, prospective, Qual (not specified) and MM (not specified) | NR | Inductive content analysis | QATSDD |
| ***Combined*** | Messina, 2017 | UK, the Netherlands, Finland, USA, Australia, Canada | 18 | 2266 | Risk of Type 2 Diabetes | diabetes prevention activities targeted at patients or clinicians | Qual (interviews, focus groups, video observation, survey), Quant (survey) and MM. | framework, thematic, thematic content, statistical, grounded theory, content | Narrative synthesis and thematic analysis | Adapted CASP checklist for qualitative and quantitative studies |
|  |  |  |  | (586 HCPs [207 GPs, 251 nurses, 45 physiotherapist, 5 allied health]; 1651 patients; 29 community leaders) |  |  |  |  |  |  |
|  | Skoglund, 2022 | Europe, Asia, South Pacific, USA, Canada, Africa | 20 | 552 patients | Pre-diabetes | Lifestyle change or maintenance (community programs or consultations with health care providers, or general diet and exercise advice) | Qual (interviews, focus groups), MM (process evaluation, interviews, observational, focus groups, conversation recordings) | discourse, content, content, grounded theory, thematic, ethnographic, IPA | Thematic synthesis | CASP checklist for qualitative research |
|  | Wandell, 2018 | UK, Canada, Netherlands, Australia, Denmark, Germany, Argentina, France, New Zealand, Singapore | 28 | 6853 HCPs | Cardiometabolic diseases (CVD, type 2 diabetes, chronic kidney disease) | selective prevention programmes | Qual (interview, focus groups, witness seminar, open-ended questions), Quant (phone survey, postal survey, observational), and MM (interviews, survey with open ended questions) | NR | Bespoke method | MMAT |
|  |  |  |  | (6536 GPs, 135 Nurses, 125 Other HCPs; 57 healthcare administrators) |  |  |  |  |  |  |
| ***Interventions to treat/manage chronic health conditions*** | | | | | | | | | | |
| ***Physical*** | Christensen, 2016 | New Zealand, USA, UK | 9 | 133 | Multiple Sclerosis | exercise and maintenance of exercise | Qual (semi-structured interviews, focus groups) | IPA, general inductive, grounded theory, analytic induction, thematic | Qualitative meta-synthesis (Sandelowski and Barroso approach) | COREQ |
|  |  |  |  | (131 patients, 2 HCPs) |  |  |  |  |  |  |
|  | Dash, 2020 | UK, Australia, Brazil, Ireland, Sweden, USA | 14 | 270 | Type 1 Diabetes | participation in physical activity | Qual and MM (interviews and focus groups) | thematic, descriptive, IPA, content | Analytical thematic analysis (Thomas and Harden approach) | CASP for qualitative research |
|  |  |  |  | (105 children or adolescents patients,108 family members, 37 teachers and 20 HCPs) |  |  |  |  |  |  |
|  | Hilberdink, 2020 | NR | 23 | 10880 (10833 patients; 47 physiotherapists) | Axial spondyloarthritis | Exercise | Quant (cross sectional, RCT, prospective), Qual (Delphi, not specified), and reviews | NR | Intervention mapping | Not assessed |
|  | Hurley, 2018 | UK, NZ, Australia, Canada, Sweden, Iceland, Netherlands | 12 | 197 patients | Osteoarthritis or chronic hip/knee pain | Exercise-based rehabilitation programmes with land or water based activities (some with education component) | Qual (interviews, focus groups, diary, survey) | Constant comparative, thematic, framework, grounded theory, general inductive, IPA, interpretational | Framework synthesis | EPPI-Centre |
|  | Kanavaki, 2017 | UK, Canada, New Zealand, Denmark, USA, Iceland, Sweden, Netherlands | 10 | 173 patients | Hip or knee osteoarthritis | Physical activity, either structured or lifestyle based | Qual studies (interviews, focus groups) | constant comparative, general inductive, thematic, framework, directed content, grounded theory, IPA, interpretational | Thematic synthesis. | CASP Qualitative Checklist |
|  | Learmonth, 2016 | NR | 19 | 235 patients | Multiple sclerosis | physical activity or exercise participation (self-directed, group exercise, resistance training, advice, strength and balance, aerobic exercise, pilates, guided home based) | Qual and MM studies (focus groups, interviews) | general inductive, IPA, thematic content, general inductive, interpretative description | Inductive coding | McMaster Critical review form |
|  | Newitt, 2016 | US, UK, Netherlands, New Zealand, Sweden, Canada | 15 | 196 patients | Various neuromusculoskeletal conditions (spinal cord injury, multiple sclerosis, cerebral palsy, Parkinsons, traumatic brain injury, motor neurone disease, muscular dystrophy, myelomeningocele, acquired brain injury and rheumatoid arthritis) | Physical activity | Qual (focus groups, interviews) | NR | Framework analysis | McMaster University Occupational Therapy Evidence-Based Practice Research Group |
|  | Tierney, 2011 | UK, USA, Sweden and Canada | 20 | 306 patients + NR carers and relatives | Heart Failure | Exercise/activity | Qual (interviews, focus group) | Thematic, content, constant comparative, IPA | Framework analysis | JBI Qualitative Assessment and Review Instrument (QARI). |
|  | Vadas, 2021 | USA, Germany, UK, Netherlands, Norway, Sweden, Singapore, India, Australia | 12 | 150 | Stroke | Exercise programs | Qual (interviews, focus group) | thematic, content, grounded theory, IPA | Thematic synthesis (Thomas and Harden approach) | CASP for qualitative studies |
|  |  |  |  | (131 patients, 10 caregivers, 9 coaches) |  |  |  |  |  |  |
| ***Psychological*** | Davenport, 2019 | UK, Brazil | 10 | 155 patients | Bipolar disorder | Psychoeducation, relapse intervention, cognitive analytic therapy, mindfulness based cognitive therapy, and novel CBT | Qual (interviews, feedback sessions) | thematic, IPA | Thematic synthesis (Thomas and Harden approach) | CASP for qualitative studies |
|  | Finazzi, 2022 | United Kingdom, Sweden, Switzerland, Norway, Ireland. | 22 | 275 patients | Mild to moderate mental health issues | CBT, mindfulness-based cognitive therapy, guided self help, Counselling for Depression, and Dynamic Interpersonal Therapy | Qual and MM (interviews, focus groups, online survey, verbal feedback) | thematic, IPA , grounded theory, framework, content, systematic text condensation, constant comparative, Foucauldian discourse | Thematic synthesis (Thomas and Harden approach) | CASP qualitative tool |
|  | Scope, 2021 | NR | 9 | 178 | Persistent physical symptoms (inclusing Chronic fatigue syndrome/myalgic encephalomyelitits, chronic low back pain, medically unexplained physical symptoms) | behavioural interventions (including symptom clinic, rehabilitation and supportive listening, cognitive functional therapy, reattribution, CBT and mindfulness stress reduction, humanistic group counselling, BodyMind Approach group intervention) | Qual or MM (interviews open ended questions, case studies) | NR | Thematic synthesis and Confidence in the Evidence from Reviews of Qualitative research (GRADECERQual) | adapted CASP for qualitative research |
|  |  |  |  | (130 patients, 48 HCPs [24 GPs, 10 physiotherapists, 8 CBT therapists, 6 nurses]) |  |  |  |  |  |  |
| ***Nutrition*** | Boocock, 2021 | UK, USA, Europe, Middle East and New Zealand. | 14 | 3046 HCPs | Type 2 Diabetes | dietary management | Qual, Quant and MM (surveys) | NR | Thematic synthesis using Framework Method and GRADE | NICE Methodology checklist for qualitative studies |
|  | Spencer, 2012 | USA | 13 | NR | Hypertension or pre-hypertension | DASH diet | NR | NR | Integrative review | Not assessed |
| ***Combined*** | Hall, 2019 | UK, Canada, New Zealand, Germany, USA, Australia, Netherlands, Ireland | 11* | 256 patients* | Low back pain | patient education on activity (advice on self-management strategies, education), refer to other HCPs (e.g.,physiotherapy, chiropractic, cognitive behavioural treatment, or pain management) | Qual (focus group, interviews) | NR | Theoretical domains framework | CASP and COREQ |
| ***Self-Management*** | Havas, 2016 | America, Australia, Canada, England, New Zealand and Singapore | 12 | 327 patients+ review of 25 studies | Chronic kidney disease | self-management support | Qual and MM (interviews, focus groups), Quant (surveys) | thematic, content, narrative summary, Heideggerian Hermeneutics | Narrative analysis | COREQ |
|  | Miles, 2017 | USA, UK, Australia, Canada, Taiwan, Denmark, Singapore, Netherlands, Germany, New Zealand, Thailand | 56 | NR (Children, adolescents and adults with asthma, carers, HCP, school staff) | Asthma | self-management (e.g., action plans, guidelines, internet and text message interventions to improve aspects of self-management; educational interventions in the form of a booklet or DVD; and medication reviews | Qual (interviews, focus groups, diary or journal, online free text, recording of clinical consultation) | NR | Thematic synthesis (Thomas and Harden approach) | CASP qualitative research |
|  | Nguyen, 2022 | US, Thailand, Singapore, Sweden, China, South Korea | 13 | 289 patients | Various (Type 2 Diabetes, Chronic Kidney Disease, hypertension, arthritis, heart failure, gout, COPD, multiple chronic diseases, asthma) | self- management | Qual or MM (focus groups, interviews) | NR | Meta- aggregation (Lockwood approach) | QARI |
|  | Othman, 2020 | US, Canada, UK | 9 | 156 | Type 2 Diabetes | Diabetes self-management | Qual or MM with separate qual data (focus group, semi-structured interviews) | Inductive, grounded theory, thematic | Thematic analysis (initial coding framework underpinned by the social-ecological theory) | CASP qualitative checklist |
|  |  |  |  | (92 patients, 56 caregivers, 8 HCPs) |  |  |  |  |  |  |
|  | Spenceley, 2006 | USA, UK, Canada, New Zealand | 16 | NR (patients) | Diabetes | Self-care | Qual, Quant, MM (not specified) | NR | Integrative review narrative synthesis | Bespoke modified qualitative and quantitative tools |
|  | Trappes-Lomax, 2016 | UK, USA, Canada, Australia, Denmark, Netherlands | 37 | 2263 | Various chronic diseases (chronic illness, diabetes, respiratory conditions, coronary heart disease, cystic fibrosis, HIV, Cancer, hypertension, back pain, atopic dermatitis, osteoarthritis, inflammatory bowel disease, anxiety, depression, menorrhagia, irritable bowel syndrome, asthma) | Self-care | Qual (interviews, focus groups, videos of consultations, diary, online content ) and Other (nested in trials, online, surveys and reviews) | thematic, framework, review, constant comparative, content, inductive thematic, meta-synthesis with meta-ethnographic approach, critical intergrative review, grounded theory | Thematic analysis by subtheme | Not assessed |
|  |  |  |  | (1805 patients, 458 HCPs) |  |  |  |  |  |  |
|  |  |  |  | Also includes unspecified participants in reviews and internet discussion boards) |  |  |  |  |  |  |
| *number of studies that data was extracted from. Abbreviations: MM = mixed methods; UK = United Kingdom; USA = United States of America; NR = Not reported; IPA = inductive phenomenological analysis; ROB = risk of bias; CASP = Critical Appraisal Skills Programme; MMAT = Mixed Methods Appraisal Tool; COREQ Consolidated Criteria for Reporting Qualitative Research; QARI = JBI Qualitative Assessment and Review Instrument; QATSDD = Quality Assessment Tool for Studies with Diverse Designs | | | | | | | | | | |
|  |  |  |  |  |  |  |  |  |  |  |
