# Supplementary figures and images for: Common barriers and enablers to the use of non-drug interventions for managing common chronic conditions in primary care: an overview of reviews
Source: BMC Prim Care. 2024 Apr 6;25:108. doi: 10.1186/s12875-024-02321-8 (PMC10998330; doi:10.1186/s12875-024-02321-8)

**Additional file 6**: Graphical Representation of Overlap (GROOVE)


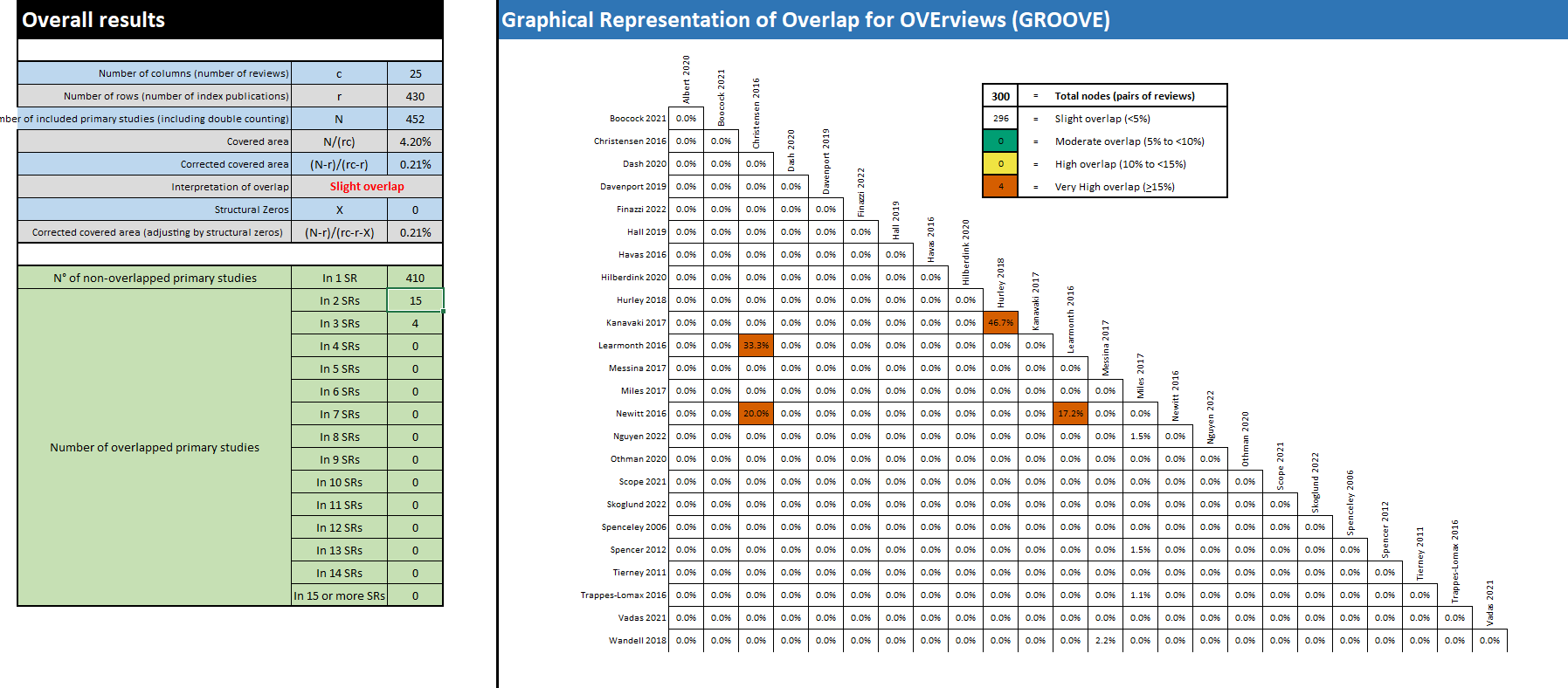

Supplement: Supplementary file 6 — Supplementary Material 6. [file 12875_2024_2321_MOESM6_ESM.docx]
