# Supplementary material for: Common barriers and enablers to the use of non-drug interventions for managing common chronic conditions in primary care: an overview of reviews
Source: BMC Prim Care. 2024 Apr 6;25:108. doi: 10.1186/s12875-024-02321-8 (PMC10998330; doi:10.1186/s12875-024-02321-8)
Supplement: Supplementary file 7 — Supplementary Material 7. [file 12875_2024_2321_MOESM7_ESM.docx]

Additional File 7: Risk of bias of primary studies

Review authors used various tools to assess RoB of primary studies, though the most common tool was Critical Appraisal Skills Program checklist for qualitative research, used by 11 included reviews. All but 3 reviews assess RoB of included studies. Of reviews that assess RoB, 12 report an overall RoB score, and 10 do not.

### Albert 2020 (52)

This included review assessed RoB using the Quality Assessment Tool for Studies with Diverse Designs (QATSDD) tool. RoB ratings for this tool can be interpreted as low (<50%), medium (50–80%), or high (>80%) quality evidence. Below is the RoB ratings for each included primary study.

| **Author, Year** | **RoB Rating** |
| --- | --- |
| Ackermann, 2005 | 81% |
| Dinan, 2006 | 39% |
| Dugdill, 2005 | 31% |
| Eynon, 2018 | 74% |
| Gademan, 2012 | 61% |
| Grandes, 2011 | 69% |
| Grandes, 2009 | 64% |
| Gusi, 2008 | 69% |
| Hanson, 2019 | 72% |
| Isaacs, 2007 | 81% |
| James, 2017 | 83% |
| Joyce, 2010 | 56% |
| Kallings, 2009 | 73% |
| Law, 2019 | 56% |
| Livingstone, 2015 | 61% |
| Lundqvist, 2017 | 69% |
| Martin-Borras, 2018 | 72% |
| Moore, 2013 | 69% |
| Patel, 2013 | 69% |
| Roessler and Ibsen, 2009 | 69% |
| Romé, 2014 | 72% |
| Sharma, 2012 | 62% |
| Shaw, 2012 | 67% |
| Sorensen, 2008 | 81% |
| Taylor, 1998 | 67% |
| Wormald & Ingle, 2004 | 44% |
| Wormald, 2006 | 51% |

### Boocock 2021 (16)

This included review assessed RoB using the NICE Methodology checklist for qualitative studies tool. RoB ratings for this tool can be interpreted as 1-3 star, with 3 star being highest rating. Below is the RoB ratings for each included primary study.

| **Author, Year** | **RoB Rating** |
| --- | --- |
| Gianfrancesco, 2020 | 3 stars |
| Jansink, 2010 | 3 stars |
| Katagiri, 2018 | 3 stars |
| Khairnar, 2019 | 3 stars |
| Hawthorne, 2012 | 2 stars |
| Hopper, 1995 | 2 stars |
| Jacques, 1993 | 2 stars |
| Marrero, 2000 | 2 stars |
| McDowell, 2012 | 2 stars |
| Parry Strong, 2014 | 2 stars |
| de Fine, 2005 | 1 star |
| Fogelman, 2015 | 1 star |
| Gross, 2008 | 1 star |
| Woodcock, 2001 | 1 star |

### Christensen 2016 (30)

This included review assessed RoB using the Consolidated Criteria for Reporting Qualitative Research (COREQ) tool. RoB ratings are not reported for this study.

### Dash 2020 (37)

This included review assessed RoB using the Critical Appraisal Skills Programmes (CASP) checklist for qualitative research tool. RoB ratings for this tool can be interpreted as a score out of 10, where higher score means higher quality.. Below is the RoB ratings for each included primary study.

| **Author, Year** | **RoB Rating** |
| --- | --- |
| Blake, 2018 | 9 |
| Burke, 2007 | 8 |
| Fereday, 2009 | 9.5 |
| Freeborn, 2013 | 7 |
| MacMillan, 2015 | 9.5 |
| MacMillan, 2016 | 8.5 |
| Mitchell, 2017 | 9 |
| Quirk, 2014 | 9 |
| Quirk, 2015 | 9 |
| Quirk, 2016 | 9.5 |
| Ryninks, 2015 | 8.5 |
| Sparapani, 2015 | 9 |
| Wennick, 2009 | 9 |
| Wilkie, 2017 | 8.5 |

### Davenport 2019 (40)

This included review assessed RoB using the Critical Appraisal Skills Programmes (CASP) checklist for qualitative research tool. Reported RoB ratings for this tool can be interpreted as S = satisfactory or U = unable to evaluate. Below is the RoB ratings for each included primary study.

| **Author, Year** | **RoB Rating** |
| --- | --- |
| Chadwick, 2011 | S |
| Evans, 2016 | U |
| Joyce, 2016 | S |
| Menezes, 2012 | U |
| Morriss, 2016 | U |
| O’Connor, 2008 | U |
| Pontin, 2009 | S |
| Poole, 2012 | U |
| Poole, 2015 | S |
| Straughan, 2006 | S |

### Finazzi 2022 (31)

This included review assessed RoB using the Critical Appraisal Skills Programmes (CASP) checklist for qualitative research tool. RoB ratings for this tool can be interpreted as high quality: 9–10; moderate quality: 7.5–8.5; low quality: 6.5–7; exclude: scores below 6. Below is the RoB ratings for each included primary study.

| **Author, Year** | **RoB Rating** |
| --- | --- |
| Allen, 2009 | 9.5 |
| Amos, 2019 | 9 |
| Bahu, 2019 | 7 |
| Bendelin, 2011 | 10 |
| Christodoulou, 2018 | 10 |
| Doukani, 2020 | 9 |
| Farrand, 2018 | 9 |
| Foy, 2019 | 7 |
| Goldman, 2016 | 8 |
| Hadfield, 2019 | 9 |
| Haller, 2019 | 9.5 |
| Holst, 2017 | 9.5 |
| Knowles, 2014 | 9 |
| Leonidaki, 2016 | 9 |
| Lillevoll, 2013 | 10 |
| Mackinnon, 2016 | 75 |
| McElvaney, 2013 | 9 |
| Millett, 2018 | 8 |
| Newbold, 2013 | 8.5 |
| Perera-Delcourt, 2019 | 9.5 |
| Rogers, 2004 | 8 |
| Rushton, 2020 | 8.5 |

### Hall 2019 (48)

This included review assessed RoB using the Critical Appraisal and Skills Programme (CASP) checklist in combination with the Consolidated criteria for reporting qualitative research (COREQ) tool. Reported RoB RoB ratings for this tool can be interpreted as good, moderate, or low methodological rigour. Below is the RoB ratings for each included primary study.

| **Author, Year** | **RoB Rating** |
| --- | --- |
| Bishop, 2015 | Moderate |
| Breen, 2007 | Moderate |
| Chenot, 2008 | Low |
| Corbett, 2009 | Good |
| Darlow, 2014 | Good |
| French, 2012 | Good |
| Fullen, 2008 | Good |
| Green, 2015 | Moderate |
| Poitras, 2012 | Good |
| Schers, 2001 | Moderate |
| Shye, 1998 | Moderate |

### Havas 2016 (53)

This included review assessed RoB using the Consolidated Criteria for Reporting Qualitative Research (COREQ) tool. RoB ratings are not reported for this study.

### Hilberdink 2020 (36)

This included review did not assess RoB of included studies.

### Hurley 2018 (51)

This included review assessed RoB using the EPPI-Centre tool. RoB ratings for this tool can be interpreted as high, medium or low credibility and dependability. Below is the RoB ratings for each included primary study.

| **Author, Year** | **RoB Rating** |
| --- | --- |
| Campbell, 2001 | High Dependability; Medium Credibility |
| Fisken, 2016 | Medium Dependability; Medium Credibility |
| Hendry, 2006 | High Dependability; High Credibility |
| Hinman, 2016 | High Dependability; High Credibility |
| Hurley, 2010 | High Dependability; High Credibility |
| Larmer, 2014b | High Dependability; Medium Credibility |
| Moody, 2012 | Medium Dependability; High Credibility |
| Morden, 2011 | High Dependability; High Credibility |
| Petursdottir, 2010 | High Dependability; High Credibility |
| Stone, 2015 | High Dependability; High Credibility |
| Thorstensson, 2006 | High Dependability; High Credibility |
| Veenhof, 2006 | Medium Dependability; High Credibility |

### Kanavaki 2017 (41)

This included review assessed RoB using the Critical Appraisal Skills Programmes (CASP) checklist for qualitative research tool. RoB ratings for this tool can be interpreted as a score out of 10 (high quality: 9–10; moderate quality: 7.5–8.5; low quality: 6.5–7; exclude: scores below 6). Below is the RoB ratings for each included primary study.

| **Author, Year** | **RoB Rating** |
| --- | --- |
| Campbell, 2001 | 6 |
| Fisken, 2016 | 6 |
| Hammer, 2015 | 6 |
| Hendry, 2006 | 9 |
| Kabel, 2014 | 6 |
| Kaptein, 2013 | 7 |
| Petursdottir, 2010 | 9 |
| Stone, 2015 | 9 |
| Thorstensson, 2006 | 7 |
| Veenhof, 2006 | 6 |

### Learmonth 2016 (33)

This included review assessed RoB using the McMaster critical review tool. RoB ratings for this tool can be interpreted as scores of 0–24, where higher score means better quality. Below is the RoB ratings for each included primary study.

| **Author, Year** | **RoB Rating** |
| --- | --- |
| Aubrey, 2012 | 16 |
| Borkoles, 2008 | 13 |
| Brown, 2012 | 11 |
| Dlugonski, 2012 | 18 |
| Dodd, 2006 | 15 |
| Elsworth, 2009 | 9 |
| Giacobbi, 2012 | 18 |
| Kasser, 2009 | 16 |
| Kayes, 2011 | 14 |
| Learmonth, 2012 | 15 |
| Normann, 2013 | 19 |
| Plow, 2009 | 16 |
| Plow, 2014 | 15 |
| Schneider, 2010 | 14 |
| Smith, 2009 | 17 |
| Smith, 2011 | 16 |
| Smith, 2013 | 13 |
| Van der Linden, 2014 | 16 |
| VanRuymbeke, 2013 | 8 |

### Messina 2017 (45)

This included review assessed RoB using the Critical Appraisal Skills Program (CASP) checklist that was adapted to suit both qualitative and quantitative studies. RoB ratings are not reported for this study.

### Miles 2017 (32)

This included review assessed RoB using the Critical Appraisal Skills Programmes (CASP) checklist for qualitative research tool. RoB ratings are not reported for this study.

### Newitt 2016 (49)

This included review assessed RoB using the McMaster University Occupational Therapy Evidence-Based Practice Research Group tool. RoB ratings for this tool can be interpreted as score out of 23, where a higher score indicates higher quality.
